# Loss of p53 suppresses replication stress-induced DNA damage in ATRX-deficient neuroblastoma

**DOI:** 10.1038/s41389-021-00363-6

**Published:** 2021-11-06

**Authors:** Jesmin Akter, Yutaka Katai, Parvin Sultana, Hisanori Takenobu, Masayuki Haruta, Ryuichi P. Sugino, Kyosuke Mukae, Shunpei Satoh, Tomoko Wada, Miki Ohira, Kiyohiro Ando, Takehiko Kamijo

**Affiliations:** 1 Research Institute for Clinical Oncology, Saitama Cancer Center, Saitama, Japan; 2 Laboratory of Tumor Molecular Biology, Department of Graduate School of Science and Engineering, Saitama University, Saitama, Japan

**Keywords:** Paediatric cancer, Oncogenes, Apoptosis

## Abstract

Genetic aberrations are present in the *ATRX* gene in older high-risk neuroblastoma (NB) patients with very poor clinical outcomes. Its loss-of-function (LoF) facilitates the alternative lengthening of telomeres (ALT) pathway in tumor cells and is strongly linked to replication stress (RS) and DNA damage through G-quadruplex (G4) DNA secondary structures. However, limited information is available on *ATRX* alteration-related NB tumorigenesis. We herein knocked out (KO) *ATRX* in *MYCN*-amplified (NGP) and *MYCN* single copy (SK-N-AS) NB cells with wild-type (wt) and truncated *TP53* at the C terminus, respectively, using CRISPR/Cas9 technologies. The loss of ATRX increased DNA damage and G4 formation related to RS in *TP53* wt isogenic *ATRX* KO NGP cells, but not in SK-N-AS clones. A gene set enrichment analysis (GSEA) showed that the gene sets related to DNA double-strand break repair, negative cell cycle regulation, the G2M checkpoint, and p53 pathway activation were enriched in NGP clones. The accumulation of DNA damage activated the ATM/CHK2/p53 pathway, leading to cell cycle arrest in NGP clones. Interestingly, ATRX loss did not induce RS related to DNA damage response (DDR) in *TP53*-truncated SK-N-AS cells. p53 inactivation abrogated cell cycle arrest and reduced G4 accumulation in NGP clones. The loss of p53 also induced G4 DNA helicases or Fanconi anemia group D2 protein (FANCD2) with ATRX deficiency, suggesting that ATRX maintained genome integrity and p53 deficiency attenuated RS-induced DNA damage in NB cells featuring inactivated ATRX by regulating DNA repair mechanisms and replication fork stability.

## Introduction

Neuroblastoma (NB) is a pediatric tumor of the sympathetic nervous system that accounts for 8–10% of all childhood cancers and 15% of pediatric oncology deaths. Genomic studies revealed that patients with high-risk NB frequently harbored recurrent *MYCN* amplification (37%), *TERT* rearrangements (23%), and alpha thalassemia mental retardation X-linked (*ATRX*) mutations or deletions (11%) [1, 2]. Mutations in *ATRX* were found to be mutually exclusive with *TERT* promoter mutations and *MYCN* amplification, and defined a distinct subgroup of older NB patients with poor outcomes [1–3]. In addition to human NB, loss-of-function (LoF) mutations in *ATRX* have frequently been detected in multiple malignancies [4–7], and have been implicated in the telomerase-independent telomere maintenance alternative lengthening of telomeres (ALT) mechanism, which has been reported in 24% of high-risk NB [8–10]. The ATRX/DAXX complex was found to be less abundant in ALT-positive NB tumors due to *ATRX* mutations (55%) or low protein expression [9], which supports the relationship between the ATRX status and ALT. Previous studies described a link between the loss of ATRX, replication stress (RS), DNA damage, copy number alterations, and genomic instability [11–15]. ATRX has more recently been suggested to bind to the G-quadruplex (G4) structure [16], with its loss leading to an increase in the G4 structure or the formation of stable DNA:RNA hybrids (R-loops), which is considered to induce replication fork stalling and collapse and the generation of DNA double-strand breaks (DSBs) at telomeres, suggesting a role for ATRX in the resolution of the G4 structure and regulation of R-loops [11, 17]. ATRX also forms a complex with DAXX to deposit H3.3, which prevents the formation of the G4 structure [11, 18, 19] or R-loops [17], thereby maintaining fork stability during acute RS.

A recent study demonstrated that somatic mutations in *TP53* pathway genes were significantly enriched in ALT-positive tumors [9]. Furthermore, p53 pathway aberrations were frequently detected in ALT NB cell lines [20]. In ALT-positive tumors, *ATRX* mutations are commonly connected to mutations in the tumor suppressor gene *TP53* [4, 21, 22], and the LoF of p53 activates the ALT pathway [20]. Collectively, these findings highlight the involvement of both *ATRX* mutations and p53 pathway aberrations in NB tumorigenesis in terms of ALT. In mouse neural progenitor cells (mNPCs), ATRX deficiency promoted p53-dependent apoptosis through the accumulation of DNA damage in the embryonic brain caused by DNA RS [15]. However, DNA damage accumulation and cell death were effectively rescued in ATRX/p53 double mutant mice [23]. Although the loss of p53 promotes the growth of emergent cancer cells by reducing RS-induced DNA damage [24], the mechanisms by which p53 deficiency suppresses RS in terms of ATRX loss remain unknown.

To avoid RS, G4 helicases prevent G4-induced genome instability by resolving G4 structures [25, 26]. Another Fanconi anemia (FA) pathway protein, FANCD2, plays a key role in limiting RS by controlling the stability of stalled replication forks in cells or tumors lacking BRAC1/2 [27, 28], and has been shown to cooperate with ATRX to limit RS and promote the homologous recombination (HR)-dependent repair of DSBs [29]. In addition to the H3.3 chaperone activity of ATRX/DAXX, the histone H3 deposition activity of FANCD2 was necessary for protecting stalled replication forks [29]. Previous studies also revealed the transcriptional regulation of G4 DNA helicases or FA pathway genes by p53, which are involved in telomere maintenance, DNA repair, and the centromere structure [30–32]. However, the impact of this regulation in ATRX-deficient cells remains unclear.

In the present study, we revealed that ATRX depletion in *TP53* wild-type (wt) NB cells was associated with an increased frequency of DSBs and a subsequent RS-induced DNA damage response (DDR), which was impaired by the loss of p53 through the activation of G4 DNA helicases or the FA DNA repair pathway protein, FANCD2. Collectively, the present results indicate that p53 deficiency limits ATRX loss-induced RS/genome integrity in NB cells by regulating DNA repair mechanisms and replication fork stability.

## Materials and methods

### Cell lines

Human NB cell lines (NGP, NB-69 and SK-N-AS) were obtained from official cell banks (RIKEN Cell Bank, Tsukuba, Japan and ATCC, Manassas, VA, USA). Additional details of cell lines are in the Supplementary Materials and Methods.

### *ATRX* CRISPR genome editing

CRISPR/Cas9 technology was used to generate *ATRX* KO cells. We designed three guide RNAs (gRNAs) against exons 4 and 5 of ATRX (Supplementary Fig. 1A, B). Further details are presented in the Supplementary Materials and Methods.

### Supplementary information

Other materials and methods and Supplementary Tables S1 to S4 are described in Supplementary Information.

## Results

### Generation of *ATRX* KO cells by CRISPR/Cas9 genome editing

**Fig. 1:**
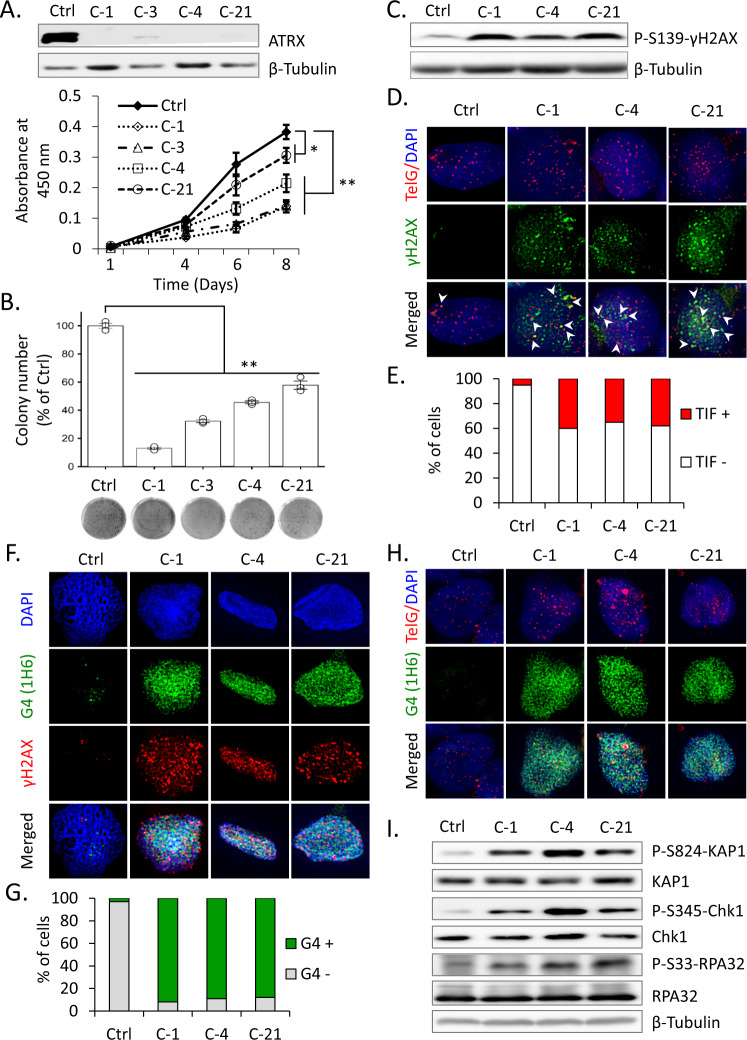
ATRX loss induces G4 formation and RS in *TP53* wt NGP cells. **A** Western blots show the depletion of ATRX protein expression in cell lysates prepared from Cas9 control (Ctrl) and *ATRX* KO (C-1, C-3, C-4, and C-21) NGP cells. β-Tubulin was used as a loading control. Lower panel, growth curves show that viability was lower in *ATRX* KO NGP cells than in Ctrl cells. Data are expressed as means ± standard deviation (SD), *N* = 3. A two-way ANOVA followed by a multiple comparison Bonferroni post hoc test was used to compare differences between groups (*p < 0.05 and **p < 0.01). **B** Clonogenic assay of Ctrl and *ATRX* KO NGP cells demonstrating the weaker proliferative abilities of KO cells than Ctrl cells. Lower panel, representative images for clonogenic formation are shown. Error bars represent SD from three technical replicates. **p < 0.01; A one-way ANOVA with Dunnett’s and Tukey’s test were used for statistical analyses. **C** Immunoblot showing activation of the DDR upon the depletion of ATRX, including the phosphorylation of histone H2AX on Ser-139 (γH2AX). **D**, **E** γH2AX/TelG immuno-FISH (**D**) shows increased TIF (telomere dysfunction-induced foci) in *ATRX* KO NGP cells. Arrows denote the colocalization of telomeric foci (red) and γH2AX signals (green). Cells were also stained with DAPI to visualize nuclei (blue). **E** Quantification of TIF+ cells among 100 cells analyzed in (**D**). **F**, **G** Coimmunofluorescence staining of *ATRX*-intact (Ctrl) and *ATRX* KO NGP cells with the anti-G-quadruplex (G4) antibody, 1H6 and anti-γH2AX. **G** Quantification of G4+ cells among 100 cells analyzed in (**F**). **H** G4 (1H6)/TelG immuno-FISH reveals colocalization of G4 on telomeric region in *ATRX* KO NGP cells. Nuclei are counterstained with DAPI (blue). **I** Representative immunoblot analysis of p-KAP1 (Ser-824), KAP1, p-Chk1 (Ser-345), Chk1, p-RPA32 (Ser-33), and RPA32, showing the activation of RS arising from ATRX deficiency. β-Tubulin served as a loading control.

We performed genome editing with the CRISPR/Cas9 system to recapitulate the cellular and molecular perspectives of ATRX deficiency in human NB using the NGP, NB-69, and SK-N-AS cell lines, which are wt for the gene. NGP and NB-69 cells have wt *TP53* with *MYCN* amplifications and *MYCN* single copy, respectively; the *MYCN* single copy SK-N-AS cell line carries a *TP53* truncation at its C terminus [33]. All three cell lines were selected for *ATRX* gene editing because neither displayed the ALT phenotype [8]; therefore, they were good comparable models for examining ATRX functions because *ATRX* is often co-mutated with *TP53* in different tumors [8, 12, 20]. Our KO cells in three cell lines were characterized in detail (Supplementary Fig. 1, “Materials and methods”). As a control, we used bulked cells (referred to as Ctrl) and two clonal cell lines (referred to as Ctrl-1 and Ctrl-2) overexpressing Cas9 alone for NGP and NB-69 or SK-N-AS cells, respectively. We isolated four independent cell clones for NGP and three clones for both NB-69 and SK-N-AS with sequence-confirmed frameshift mutations in *ATRX* (Supplementary Fig. 1C) and completely devoid of ATRX protein expression, except for KO (C-3) NGP cells (Fig. 1A, Supplementary Figs. 2A, B, 3A and Fig. 4A). ATRX protein expression in *ATRX* KO cells for NGP and SK-N-AS were also verified using immunofluorescence (IF) (Supplementary Fig. 2A, B). Therefore, we successfully established several *ATRX* KO isogenic cells in *TP53* wt NGP, NB-69, and *TP53* truncated SK-N-AS cell lines for further study.

### ATRX loss induces G4 formation and RS in *TP53* wt NB cells

To clarify the biological consequences of ATRX deficiency, we initially examined the viability and clonogenic survival of *ATRX* KO NGP and NB-69 cells. An assessment of cell viability using the WST-8 assay showed that ATRX loss resulted in failed cell proliferation (Fig. 1A, Supplementary Fig. 3A), which is consistent with previous findings on ATRX deficiency in mNPCs and glioblastoma cells [12, 13]. We also observed lower clonogenic survival in *ATRX* KO than in Ctrl cells (Fig. 1B, Supplementary Fig. 3B).

ATRX plays a key role in the regulation of DNA replication and DNA damage repair pathways [13, 14, 34]. To ascertain whether ATRX depletion results in the accumulation of DNA damage at telomeres, we investigated γH2AX levels as a marker of stalled replication forks and DSBs. γH2AX levels were elevated in *ATRX* KO cells (Fig. 1C, Supplementary Fig. 3C), suggesting increases in stalled and collapsed replication forks. We also found enhanced telomeric DDR in *ATRX* KO NGP cells, as indicated by the increased formation of γH2AX-associated telomere dysfunction-induced foci, TIF (Fig. 1D, E). This result suggests that ATRX also functions to protect against telomere DNA damage for telomere maintenance.

We then investigated whether increased levels of DNA damage under ATRX-deficient conditions were induced by the formation of G4. Consistent with previous findings [11, 35], G4 formation at stalled replication forks was associated with RS and DDR. Using a monoclonal antibody that recognizes the G4 structure in situ (1H6), the nuclear accumulation of G4 was found to be higher in *ATRX* KO cells than in Ctrl cells (Fig. 1F, G and Supplementary Fig. 3D-E). Moreover, G4s more extensively colocalized with DNA damage foci compared with telomere region in the setting of ATRX deficiency (Fig. 1F, H and Supplementary Fig. 3D). The specificity of 1H6 antibodies for the G4 structure has already been confirmed [11]. These results suggest that ATRX deficiency induces G4 formation, indicating a role for ATRX in resolving the G4 structure at stalled replication forks.

To clarify whether increased G4 levels result in RS in *ATRX* KO NGP cells, we performed a Western blot analysis of RS signaling pathways. As shown in Fig. 1I, ATRX loss increased phospho-KAP1, phospho-Chk1, and phospho-RPA32 levels. Furthermore, IF showed that ATRX loss induced phospho-RPA32 foci (Supplementary Fig. 4A) and colocalization with G4 signal (Supplementary Fig. 4B), suggesting that ATRX is required to limit RS. Collectively, these results indicate that ATRX deficiency promoted RS and DDR in *TP53* wt NB cells.

Recent studies reported that ATRX deficiency promote the ALT phenotype by inducing HR, which is exhibited by tumors harboring *ATRX* mutations [13, 19]. To assess the effects of ATRX loss on ALT in NB cell lines, we examined ALT-associated features in *ATRX* KO NGP (Supplementary Fig. 5A-D) and NB-69 cells (data not shown). The hallmarks of ALT, namely, C-circles (Supplementary Fig. 5B) and ALT-associated PML bodies (APBs) (Supplementary Fig. 5C, D), were not induced after the loss of ATRX in these KO cells. Moreover, overall telomeric DNA between Ctrl and *ATRX* KO cells was unchanged (Supplementary Fig. 5A). Therefore, ATRX deficiency itself was not associated with the ALT phenotype in *ATRX* KO *TP53* wt NB cells, which is consistent with previous findings.

### Increased DNA damage induces the ATM/CHK2/p53 pathway in *TP53* wt *ATRX* KO NB cells

**Fig. 2:**
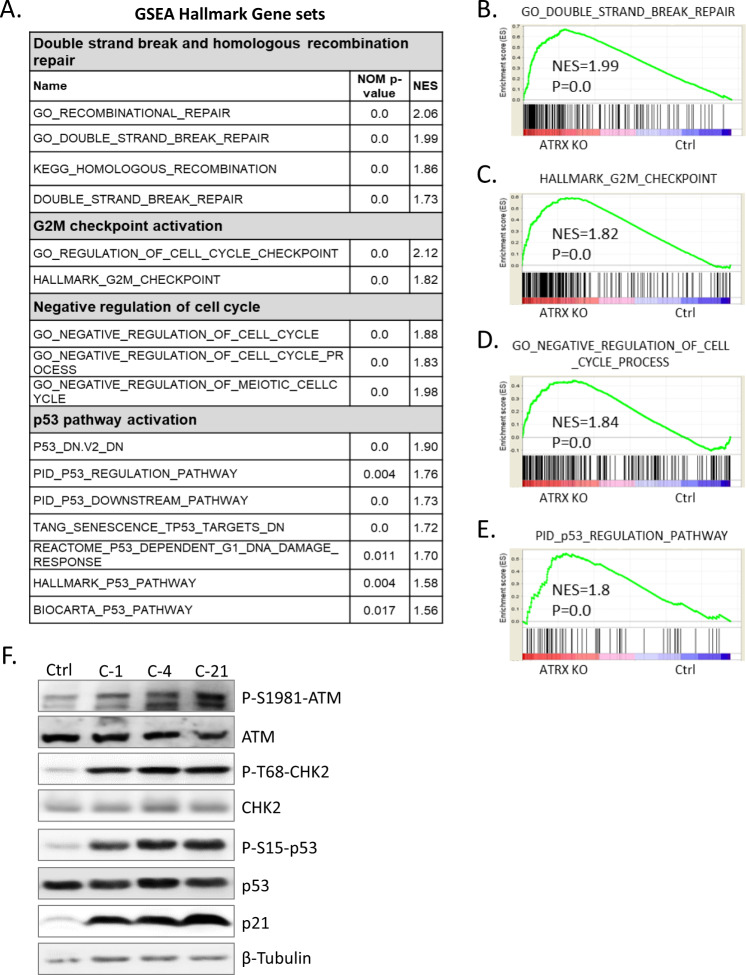
Increased DNA damage leads to ATM/CHK2/p53 pathway induction in *ATRX* KO NGP cells. **A** The Gene Set Enrichment Analysis (GSEA) results of differentially expressed gene sets between Ctrl and *ATRX* KO NGP cells. Collected nominal p-values and normalized enrichment scores (NES) were listed. **B**–**E** GSEA enrichment plot. The gene sets of **B** ‘GO DOUBLE STRAND BREAK REPAIR’, **C** ‘HALLMARK G2M CHECKPOINT’, **D** ‘GO NEGATIVE REGULATION OF CELL CYCLE PROCESS’, and **E** ‘PID p53 REGULATION PATHWAY’ were significantly enriched in *ATRX* KO NGP cells. **F** Representative immunoblots for the expression of the indicated proteins in cell lysates prepared from Ctrl and *ATRX* KO NGP cells. *ATRX* KO NGP cells showed the increased activation of p53-ATM checkpoint proteins. β-Tubulin was used as a loading control.

To elucidate ATRX-dependent transcriptional alterations and their functional consequences, we performed a microarray analysis of Ctrl and *ATRX* KO NGP cells. According to GSEA, upregulated genes were implicated in DSBs and HR repair, cell cycle checkpoint activation, negative cell cycle regulation, and p53 pathway activation (Fig. 2A–E). This result suggested that the pathways involved in negative cell cycle regulation and DNA damage-induced p53 pathway activation were enhanced by ATRX deficiency in *TP53* wt NGP cells accompanied by transcriptional changes in their related gene members.

Proliferation inhibition by ATRX deficiency may be mediated by increases in DNA damage and activation of the ATM/CHK2/p53 pathway, supporting GSEA results. In response to DNA damage, p53 is phosphorylated by the ATM/CHK2 pathways, leading to cell cycle arrest or apoptosis [36]. Therefore, we investigated the involvement of ATM signaling in response to increased DNA damage. A western blot analysis of Ctrl and *ATRX* KO NGP cells demonstrated an increase in the activation of ATM in *ATRX* KO cells (Fig. 2F). ATR activation was not observed in Ctrl or *ATRX* KO cells (data not shown). Moreover, consistent with the formation of γH2AX in *ATRX* KO cells, the activation of ATM-dependent DSB signaling events was also detected in these cells, as revealed by increases in the phosphorylation of CHK2 and p53. Activated p53 then upregulated the expression of p21, which mediated the inhibition of proliferation. Moreover, p53 activation was observed in *ATRX* KO NB-69 cells, but downstream γH2AX and p21 only modest (Supplementary Fig. 3C). Collectively, these results revealed that the loss of ATRX in both *MYCN*-amplified and *MYCN* single copy *TP53* wt NB cells promoted the accumulation of DNA damage and activated the cell cycle checkpoint pathway of DDR, the ATM-CHK2-p53-p21 pathway, leading to cell cycle arrest.

### p53 inactivation limits ATRX loss-induced G4 formation and RS in *TP53* wt NGP cells

**Fig. 3:**
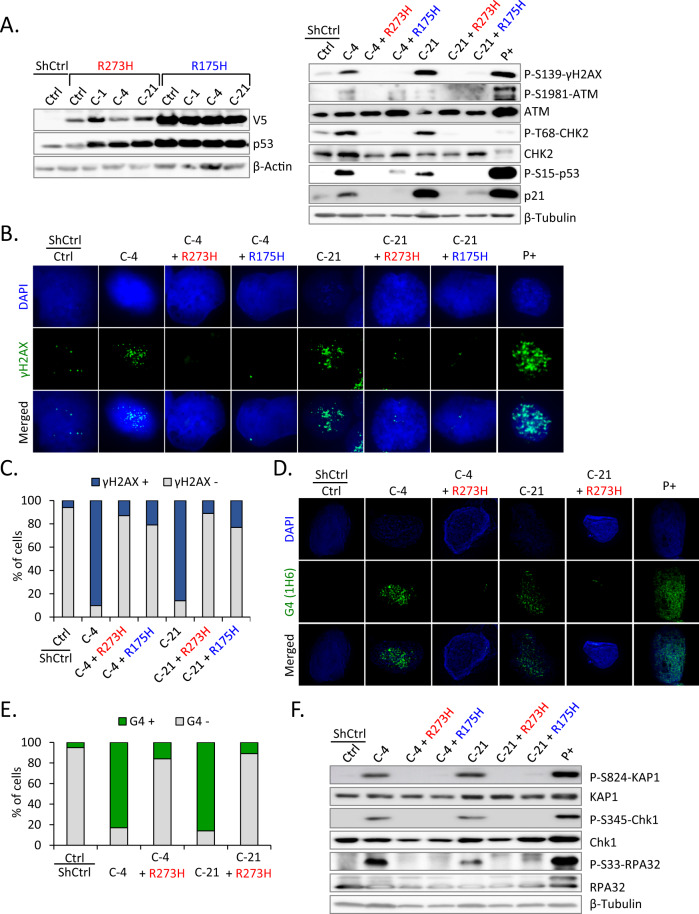
p53 inactivation limits ATRX loss-induced G4 formation and RS in *TP53* wt NGP cells. **A** Left panel, for *p53* inactivation, C-terminally V5-tagged dominant-negative p53 mutants (p53_R273H or p53_R175H) or a control empty vector (ShCtrl) were stably introduced by lentiviral infection into Ctrl or *ATRX* KO NGP cells. Western blotting using anti-V5 and anti-p53 antibodies analyzed the expression levels of V5-tagged p53 mutants. β-Actin was used as a loading control. Right panel, immunoblots for the expression of DSB checkpoint proteins in cell lysates prepared from the indicated samples. p53 inactivation in *ATRX* KO NGP cells results in the loss of the DDR and p53-ATM checkpoint proteins. β-Tubulin was used as a loading control. As a positive control (P+), parental NGP cells were treated with doxorubicin (0.5 μg/mL) for 24 h. **B**, **C** γH2AX IF indicates decreased DDR after p53 inactivation in *ATRX* KO NGP cells. NGP cells treated with doxorubicin (0.5 μg/mL, 24 h) were used as the positive control. **C** Quantification of γH2AX+ cells among 100 cells analyzed in (**B**). **D**, **E** IF staining of G4 (1H6) in the indicated cells. As a positive control (P+), parental NGP cells were treated with the DNA G-quadruplex stabilizer CX-5461 (50 nM) for 24 h and stained with the anti-G4 (1H6) antibody. Nuclei are counterstained with DAPI (blue). **E** Quantification of G4+ cells among 100 cells analyzed in (**D**). **F** The RS markers p-KAP1, p-Chk1, and p-RPA32 were not induced in *p53*-inactivated *ATRX* KO NGP cells, representing a reduction in RS. β-Tubulin was used as a loading control.

The inactivation of p53 by a viral oncoprotein or *TP53* mutation is commonly observed in ALT-positive cell lines [37]; ALT phenotype in NB is associated with *ATRX* mutation and p53 pathway alterations [20]. To establish whether p53 deficiency with the loss of ATRX promotes ALT in NB cells, a C-terminally V5-tagged dominant-negative p53 (p53_R273H or p53_R175H) vector or control was stably transduced into *ATRX* KO NGP cells (Fig. 3A, left panel). Mutant p53 exerts a dominant-negative effect by preventing wt p53 from binding to the promoter of its target genes [38]. We initially investigated whether the forced expression of mutant p53 affected the survival of *ATRX* KO NGP cells. The results obtained showed that it significantly enhanced cell survival during cell cultures, indicating that mutant p53 reduced the ability of wt p53 to induce cell cycle arrest in *ATRX* KO NGP cells (Supplementary Fig. 6). The hallmarks associated with ALT were then examined in those cells. Similar to original *ATRX* KO NGP cells, p53_R273H or p53_R175H did not display C-circles (Supplementary Fig. 7B) or APBs (Supplementary Fig. 7C, D). Moreover, the total telomeric DNA content did not significantly differ between Ctrl and *ATRX* KO cells with the p53 mutant (Supplementary Fig. 7A). Therefore, NGP cells with ATRX deficiency and the p53 status cannot induce the ALT phenotype, which is associated with *ATRX* mutation and p53 pathway aberration in NB.

An investigation of DDR in p53-inactivated *ATRX* KO NGP cells revealed that the activation of the ATM/CHK2/p53 pathway decreased after p53 inactivation. The levels of key phosphoproteins involved in DDR were lower in *ATRX* KO NGP cells with the p53 mutant than in original KO cells (Fig. 3A, right panel). IF of γH2AX also showed a weaker signal in p53-inactivated *ATRX* KO NGP cells (Fig. 3B, C), suggesting the influence of the p53 status on DDR-related cell cycle arrest upon the loss of ATRX. p53 inactivation abolished DDR and also prevented the formation of G4 (Fig. 3D, E) and suppressed the RS pathway in *ATRX* KO NGP cells (Fig. 3F). Furthermore, IF showed that p53 inactivation reduced phospho-RPA32 foci (Supplementary Fig. 8), consistent with immunoblot results. Therefore, p53 deficiency decreased ATRX loss-induced RS and DDR.

### ATRX deficiency-related DDR and RS are not induced in *TP53* truncated (C terminus) SK-N-AS cells

We attempted to confirm the ALT phenotype or p53 deficiency-related suppression of RS and DDR upon the loss of ATRX in the NB cell line with mutant *TP53* and a *MYCN* single copy. A previous study reported an ALT phenotype associated with *ATRX* mutations, the lack of *MYCN* amplification, and p53 pathway alterations in NB [20]. Therefore, we selected the SK-N-AS cell line carrying a *MYCN* single copy and *TP53* truncation at the C terminus [33]. We isolated several isogenic *ATRX* KO clones from SK-N-AS cells (discussed in the “Materials and methods” and “Results” sections). We then assessed the ALT status and found that the total telomere content and C-circle levels were below the ALT cut-off value (Supplementary Fig. 9A, B). However, APBs were partially detected in *ATRX* KO SK-N-AS cells (Supplementary Fig. 9C, D). Although APBs are one of the important markers of the ALT phenotype, C-circles are very specific and quantifiable markers of ALT [39]. Therefore, true ALT features were not observed in SK-N-AS cells after *ATRX* KO. These results suggest that, at least under the present experimental conditions, the ALT phenotype in NB cell lines was not associated with *ATRX* mutations, the lack of *MYCN* amplification, or p53 pathway aberrations.

**Fig. 4:**
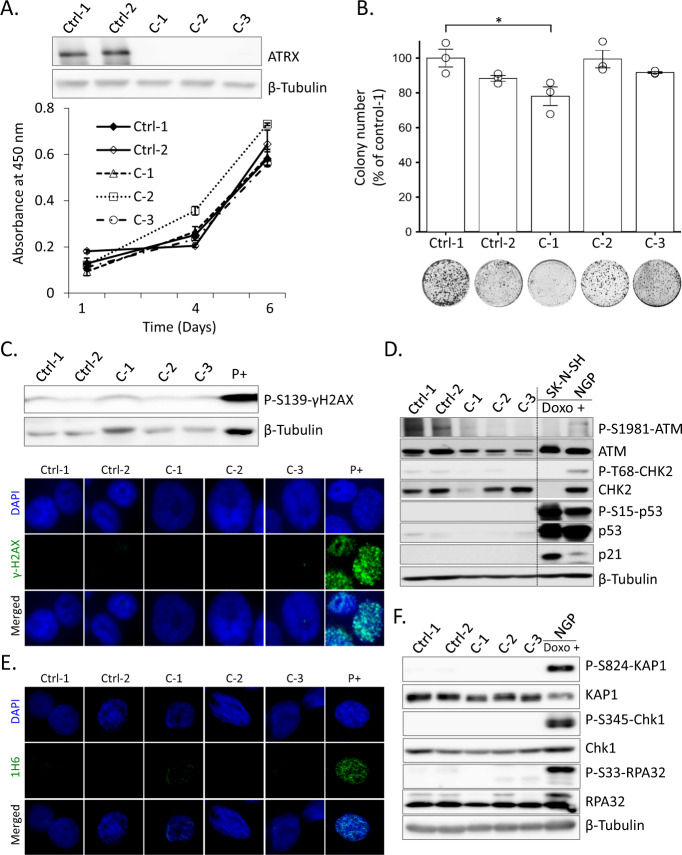
ATRX deficiency-related DDRs and RS were not induced in *TP53*-truncated (C terminus) SK-N-AS cells. **A** Western blot to confirm ATRX ablation in Cas9 control (Ctrl) and *ATRX* KO (C-1, C-2, and C-3) SK-N-AS cells. Growth curves show that cell viability was unchanged between Ctrl and *ATRX* KO SK-N-AS cells. Data are expressed as means ± standard deviation (SD), *N* = 3. **B** Colony formation assays were performed to assess the proliferative abilities of Ctrl and *ATRX* KO SK-N-AS cells. Lower panel, representative images for clonogenic formation. Error bars represent SD from three technical replicates. *p < 0.05; A one-way ANOVA with Dunnett’s and Tukey’s test were used for statistical analyses. **C** Immunoblot and IF analyses of γH2AX in *ATRX*-intact (Ctrl) and *ATRX* KO SK-N-AS cells with a positive control (parental SK-N-AS cells treated with doxorubicin, 0.5 μg/mL for 24 h). **D** Immunoblots for the expression of p53-ATM checkpoint proteins in cell lysates prepared from Ctrl and *ATRX* KO SK-N-AS cells. Doxorubicin (0.5 μg/mL)-treated two NB cell lines (SK-N-SH and NGP) were used as a positive control. **E** IF staining of G4 (1H6) revealed that ATRX deficiency did not cause G4 formation in *ATRX* KO SK-N-AS cells. The DNA G-quadruplex stabilizer CX-5461 (50 nM, 24 h) was used as a positive control to stain G4 (1H6). **F** Whole-cell extracts from Ctrl and *ATRX* KO SK-N-AS cells were analyzed by western blotting with the p-KAP1 (Ser-824), KAP1, p-Chk1 (Ser-345), Chk1, p-RPA32 (Ser-33), and RPA32 antibodies. β-Tubulin was used as a loading control. Doxorubicin (0.5 μg/mL, 24 h)-treated NGP cells were used as a positive control.

WST-8 and colony formation assay results showed that the loss of ATRX did not significantly alter the viability of SK-N-AS cells (Fig. 4A, B). Moreover, γH2AX foci were not detected in *ATRX* KO SK-N-AS cells, indicating low levels of DNA damage (Fig. 4C) which is contrast with others findings following *ATRX* KO in *TP53* wt SK-N-SH cells with prior p53 inactivation [34]. These relative phenotypic differences may be due to different experimental strategy or cell line specificity. As expected, ATRX deficiency did not induce the ATM/CHK2/p53 pathway (Fig. 4D). In addition, G4 formation was not detected in KO cells, leading to a suppressed RS response (Fig. 4E, F). In contrast to previous findings [15], these results indicated that p53 aberrations inhibited ATRX loss-induced RS related to DDR in NB cells.

### p53 inactivation leads to the upregulation of G4-resolving helicases and FA pathway proteins involved in replication fork protection

**Fig. 5:**
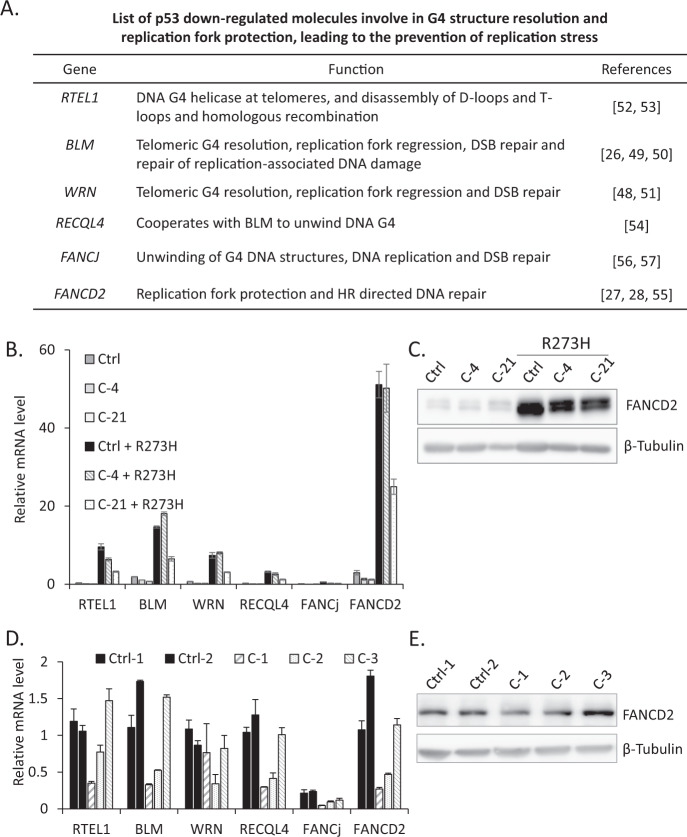
p53 inactivation leads to the upregulation of G4-resolving helicases and FA pathway proteins involved in replication fork protection. **A** List of p53 downregulated G4-resolving eukaryotic helicases or FA pathway DNA repair molecules and their proposed biological functions during DNA replication. **B** A comparison of *TP53* wt and p53-inactivated Ctrl or *ATRX* KO cells suggested the p53-dependent regulation of G4-resolving helicases and the FA pathway DNA repair molecule, FANCD2. Real-time RT-PCR analysis of *RTEL1*, *BLM*, *WRN*, *RECQL4*, *FANCJ*, and *FANCD2* in the indicated samples. Relative expression normalized to GAPDH, mean ± SD (*n* = 3). **C** p53 inactivation leads to increased FANCD2 protein levels. Protein extracts, prepared from *TP53* wt and p53-inactivated Ctrl or *ATRX* KO NGP cells, were immunoblotted with antibodies against FANCD2 and β-Tubulin. **D** Real-time RT-PCR analysis of G4-resolving helicase genes and *FANCD2* in Ctrl and *ATRX* KO SK-N-AS cells. Relative expression normalized to GAPDH, mean ± SD (*n* = 3). **E** Western blot analysis of FANCD2 in Ctrl and *ATRX* KO SK-N-AS cells. β-Tubulin served as a loading control.

When replication forks encounter DNA damage, G4 or R-loops may hinder fork progression and increase RS [40]. Under these conditions, specific proteins are recruited to the site of the stalled fork in order to manage the cellular response to this stress. Recent studies described an emerging class of DNA unwinding enzymes, known as helicases, which directly resolve the G4 DNA structure and remove RS during DNA replication [25, 26]. Another well-studied G4 helicase regulator or FA pathway protein, FANCD2, stabilizes and helps to restart stalled replication forks, thereby avoiding the generation of DNA damage and genome instability [27, 28]. To directly investigate how the p53 status affects ATRX loss-mediated RS-induced DSBs, we searched for p53-regulated molecules involved in G4 structure resolution and replication fork protection, leading to the release of RS (Fig. 5A) [30–32]. We speculated that in the absence of ATRX, other G4-resolving helicases or DNA repair molecules may prevent RS during DNA replication. Accordingly, our initial aim was to clarify whether the inactivation of p53 triggered the upregulation of these molecules in Ctrl or *ATRX* KO NGP cells. Therefore, we compared the mRNA levels of six candidate genes implicated in G4 structure resolution and replication fork recovery in *TP53* wt and p53-inactivated Ctrl or *ATRX* KO NGP cells. Five out of the six genes were upregulated after the inactivation of p53 (*RTEL1*, *FANCD2*, *BLM*, *WRN*, and *RECQL4*) (Fig. 5B), which is consistent with previous findings on p53-dependent regulation [30–32]. Importantly, the induction of *FANCD2* expression by p53 inactivation was the strongest. In addition to FANCD2, *BLM* expression also increased (Fig. 5B). These results appear to support previous findings showing that FANCD2 cooperated with BLM to facilitate stalled fork restart and suppress new origin firing; FANCD2 and BLM were also shown to have cooperative as well as independent roles in the context of stalled fork recovery [41]. Moreover, FANCD2 protein levels were higher in p53-inactivated Ctrl or *ATRX* KO cells than in *TP53* wt Ctrl or *ATRX* KO NGP cells, which was consistent with qPCR data (Fig. 5B, C). These results support the previous finding of FANCD2 expression being markedly affected by the activation of p53 [30].

We also examined the expression of G4 helicases and *FANCD2* at the mRNA level in Ctrl and *ATRX* KO SK-N-AS cells (Fig. 5D). An immunoblot analysis showed that FANCD2 protein levels remained unchanged between Ctrl or *ATRX* KO cells (Fig. 5E), suggesting that basal FANCD2 levels in SK-N-AS cells were sufficient for replication fork protection after the loss of ATRX.

Furthermore, ALT-positive NB cell lines with the *TP53* mutation (SK-N-FI) had higher *FANCD2* and *BLM* expression levels than *TP53* wt (NGP) and *TP53*-truncated (SK-N-AS) cells (Supplementary Fig. 10A). To date, no functional assays have been performed to establish whether these phenomena are the result of *TP53* mutation in ALT-positive NB cells with ATRX LoF. In public datasets (R2, http://r2.amc.nl), *FANCD2* expression levels were shown to be strongly associated with an advanced tumor stage and poor prognosis in human NB (Supplementary Fig. 10B, C). These findings suggest that upregulated *FANCD2* expression may promotes the ALT phenotype in *ATRX*-mutated NB. Collectively, these results indicate that FANCD2 expression levels significantly increased under the setting of p53 deficiency and may cooperate in the protection of stalled replication forks after the loss of ATRX.

### p53 deficiency limits ATRX loss-induced RS and genome instability through the FA pathway protein, FANCD2

**Fig. 6:**
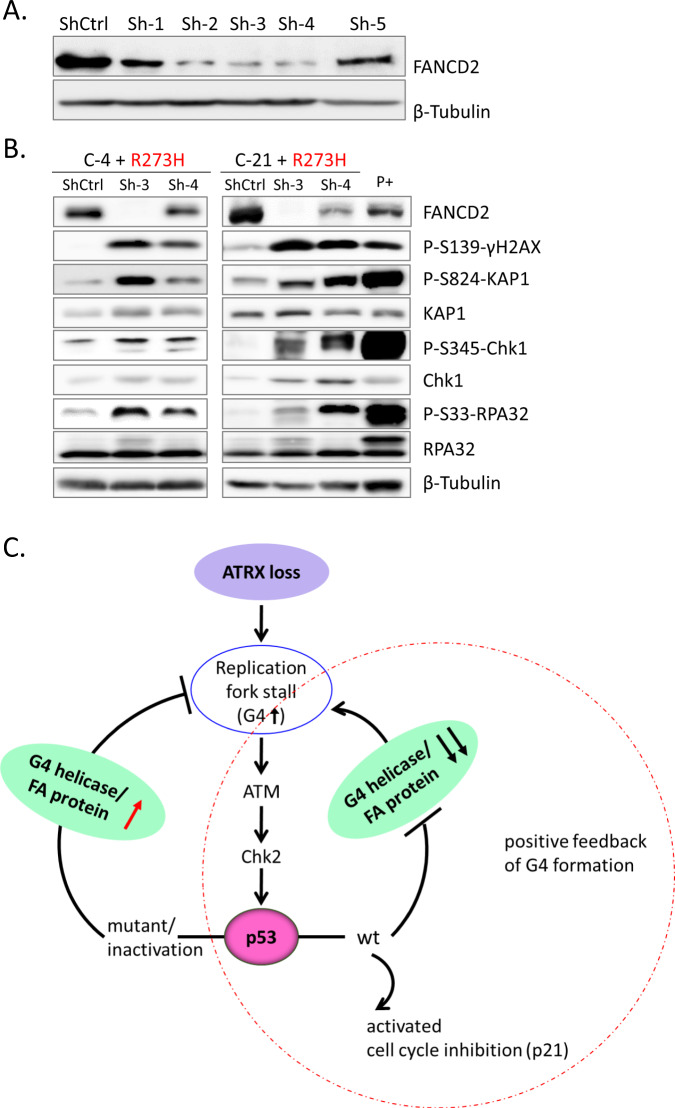
p53 deficiency limits ATRX loss-induced RS and genome instability through the FA pathway protein, FANCD2. **A** A western blot analysis showing the silencing efficiency of shRNAs against FANCD2 in HeLa cells. **B** FANCD2 protein depletion by shRNA was confirmed by immunoblotting. FANCD2 knockdown in p53-inactivated *ATRX* KO NGP cells resulted in the further activation of the DDR and RS. NGP cells treated with doxorubicin (0.5 μg/mL, 24 h) were used as a positive control. **C** Model for linking ATRX, p53, and replication fork-protecting molecules (G4 helicases or the FA pathway protein, FANCD2) to the DDR, RS, and genomic instability. G4 helicases or FANCD2 activation protected cells from RS in p53-inactivated ATRX-deficient cells. In *TP53* wt cells, the loss of ATRX resulted in DNA damage and RS in response to replication fork stalling. p53 inactivation inhibited replication fork stalling by triggering G4-resolving helicases and FANCD2 expression, which may promote G4 structure resolution and replication fork protection.

The loss of ATRX hinders fork stability by G4 or R-loop formation and increases RS (Fig. 1, Supplementary Fig. 3) [11, 17]. Since ATRX and FANCD2 have recently been reported to cooperate in replication fork recovery [29], we investigated whether FANCD2 decreases RS in the context of ATRX deficiency. We validated the knockdown efficiency of several FANCD2 shRNAs in HeLa cells and found that shRNA 3 and shRNA 4 efficiently knocked down FANCD2 at the protein level (Fig. 6A). We then measured the RS marker in two p53-inactivated *ATRX* KO NGP cells infected with FANCD2 shRNA. In cells lacking FANCD2 and ATRX, we observed elevated levels of γH2AX, which marks DNA damage sites, as well as stronger RS signals (Fig. 6B). Therefore, FANCD2 was required to prevent RS and ultimately genomic instability in ATRX-deficient cells, similar to BRCA2 deficiency [27, 28]. Cell proliferation assays showed that the inhibition of FANCD2 significantly decreased the survival of p53-inactivated *ATRX* KO NGP cells (Supplementary Fig. 11A, B). In addition, FANCD2 downregulation reduces the cell viability of ALT-positive SK-N-FI cells (Supplementary Fig. 12A-C). Therefore, FANCD2 may be partly required for the survival of ATRX-deficient cells, suggesting a synthetic lethal relationship between them. Collectively, these results propose a key role for FANCD2 in the protection of replication forks upon the loss of ATRX in NB cells.

## Discussion

In the present study, ATRX deficiency activated RS and DDR through the G4 formation, leading to cell cycle arrest in *TP53* wt NB cells. Both *MYCN* single and *MYCN* amplification may activate p53 pathway in *ATRX* KO cells and drive RS. The synthetic lethal relationship between *ATRX* mutation and *MYCN* amplification has been reported [3]. However, *TP53*-truncated SK-N-AS cells were devoid of the DDR pathway upon the loss of ATRX. Moreover, the inactivation of p53 by dominant p53 mutants (R273H and R175H) in *ATRX* KO NGP cells rescued cell viability in response to the inhibition of ATM/CHK2/p53 pathway activation. However, the mechanisms by which the p53 status influences the effects of ATRX deficiency on the fate of NB cells currently remain unknown. Therefore, we initially demonstrated that p53 deficiency limited ATRX loss-induced RS/genome integrity in NB cells by regulating G4 helicases and the replication fork-protecting FA pathway protein, FANCD2 (Fig. 6C).

Approximately 24% of high-risk NB harboring the ALT phenotype and 50–55% of ALT NB have somatic alterations in *ATRX* [1, 2, 8, 9]. ALT is a HR-based mechanism, and ATRX deficiency induced the formation of G4 in the GC-rich regions of DNA, leading to replication fork stalling and the provision of a substrate for HR [11, 42]. The loss of ATRX also promoted the accumulation of R-loops at telomeres [17], which facilitate the formation of G4 structures in untranscribed DNA strands [43]. Moreover, replication fork stalling and collapse may generate DSBs and DNA damage pathway signaling in *ATRX*-mutant glioma. While the loss of ATRX induces RS and DNA damage via G4 DNA, ATRX deficiency in conjunction with the chemical stabilization of G4 enhanced RS to induce DNA damage and cell death [11]. Consistent with these findings, we also observed G4 formation, RS, and DDR leading to cell cycle arrest as a consequence of ATRX deficiency in *TP53* wt NB cells, supporting the findings that ATRX-negative NPCs induce excessive levels of DNA damage in the embryonic brain caused by DNA RS [15]. Moreover, the ATM/CHK2/p53 pathway was found to be activated in response to ATRX deficiency-associated DNA damage, indicating that ATRX-null cells mostly depend on ATM-associated DDR signaling pathways for DNA repair [15, 44], which is consistent with other findings [45]. p53 inactivation or mutant *TP53* with ATRX deficiency inhibited the ATM/CHK2/p53 pathway, which may be attributed to the inhibited formation of G4.

Emerging studies revealed that cancer cells with ALT were hypersensitive to the inhibition of ATR, another component of DNA damage checkpoint-activating kinases other than ATM in human cells [46]. In contrast, in several telomerase-positive and ALT-positive NB or non-NB cell lines treated with an ATR inhibitor, ALT-positive cells were not generally more sensitive to ATR inhibition than telomerase-positive cells [34, 47]. We also did not observe the significant activation of ATR in NB cells featuring the inactivation of ATRX, which appears to support the finding that differences in ATR inhibitor sensitivity were not related to ATRX-deficient ALT [47].

While p53 inactivation rescued the survival of ATRX-deficient cells, in an attempt to identify the underlying survival mechanism, we focused on G4 helicases and their regulator, which is involved in the protection of replication forks. We hypothesized that in the absence of ATRX, other molecules may contribute to overcoming the G4 or R-loop secondary DNA structure and promoting stalled replication fork recovery, which are negatively regulated by p53. A number of p53 downregulated G4-interacting helicases that are capable of unfolding G4 structures have been reported [30–32]. They include the RECQ helicase family, WRN, BLM, RECQL4, or RTEL1. WRN and BLM play major roles in unwinding G4 DNA during telomere replication [25, 26, 48–51]; however, RTEL1 has also been implicated in G4 processing and is involved in DNA replication and recombination or required for the maintenance of telomere integrity [52, 53]. RECQ helicases are strongly expressed in various cancers and their overexpression provides a survival advantage to cancer cells through the protection of stalled replication forks against breakage and possibly the restart of broken replication forks. Therefore, RECQ helicases have been proposed as a target for anticancer therapy because the inhibition of RECQ helicases induced DSBs in different origins and reduced the proliferation of cancer cell lines [48, 54]. In addition to RECQ helicases, p53 also downregulated FA DNA repair pathway proteins, such as FANCD2 and FANCJ, which are known to be involved in replication fork stabilization or the resolution of R-loops by recruiting RNA processing factors [30, 55] and G4 structures [48, 56, 57], respectively. Moreover, G4 helicases in complex with FA proteins collaborate in response to stalled replication forks [58]. In our targeted screening, *FANCD2*, *BLM*, *WRN*, *RTEL1*, and *RECQL4* mRNA expression were increased in *ATRX* KO NGP cells after the inactivation of p53. *FANCD2* and *BLM* expression was strongly induced by the inactivation of p53. Moreover, we showed that the inhibition of FANCD2 further induced DNA DSB-related RS, thereby decreasing the cellular growth of p53-inactivated *ATRX* KO NGP and ALT-positive SK-N-FI cells. Consistent with previous findings, FANCD2 in the present study may also appeared to stabilize replication forks [28] and restart forks by cooperating with BLM helicase [41, 58]. FANCD2 also promoted the MRE11 exonuclease-dependent restarting of forks through the deposition of a histone H3 variant, which is a crucial event during the re-initiation of DNA replication and recruitment of CtIP at stalled replication forks [29].

In contrast, the upregulation of FANCD2 was positively associated with tumor size and a poor prognosis in breast cancer, ovarian cancer, nasopharyngeal carcinoma, glioblastoma, endometrial carcinoma, and esophageal squamous cell carcinoma [59–65]. Similarly, FANCD2 was found to be upregulated in advanced stage NB, and Kaplan–Meier survival curves showed a worse prognosis in patients with high FANCD2 expression levels (R2 database). These findings suggest that the strong expression of FANCD2 may promotes the ALT phenotype in *ATRX*-mutated NBs, and supports other findings showing that FANCD2 was necessary for telomere maintenance in ALT cells by facilitating the formation of C-circles, possibly by promoting the recruitment of BLM to replication-stressed ALT telomeres [66]. A recent study also described the non-canonical function of FANCD2 with several nuclear receptors to regulate the ALT telomere maintenance pathway [67]. In addition to *FANCD2* and *FANCJ*, other FA genes, including *FANCM*, were negatively regulated by p53 [30]. FANCM is a FA helicase that is essential for the viability of ALT cancer [68]. FANCD2 is required for the functional consequences of FANCM in response to DDR [69]. The inhibition of FANCM was previously shown to induce a potent acute apoptotic phenotype in ALT cancer cell lines [68], suggesting its potential as an anticancer target in ALT. Therefore, as a regulator of FANCM, FANCD2 will be an excellent target candidate for the treatment of ALT cancers.

The present results demonstrated the dependency of ATRX-deficient cells on p53 dysfunction for cellular survival. Moreover, we proposed that the dependency by these cells on the loss of p53 is mediated by G4 or R-loop resolution and replication fork stability as well as the recovery function of G4 helicases and the FA pathway protein, FANCD2. The present results may also expand the previously suggested roles of FANCD2 as a master regulator/handler of endogenous RS, thereby representing a therapeutic target for ATRX-deficient tumors. Further studies are needed to clarify the mechanisms by which ATRX deficiency interacts with the loss of p53 for NB progression and therapeutic responses.

## Supplementary information

supplementary information
