# Supplementary material for: Loss of p53 suppresses replication stress-induced DNA damage in ATRX-deficient neuroblastoma
Source: Oncogenesis. 2021 Nov 6;10(11):73. doi: 10.1038/s41389-021-00363-6 (PMC8572175; doi:10.1038/s41389-021-00363-6)
Supplement: Supplementary file 1 — supplementary information [file 41389_2021_363_MOESM1_ESM.pdf]

Supplementary information

Loss of p53 suppresses replication stress-induced DNA damage in ATRX-deficient neuroblastoma

Jesmin Akter, Yutaka Katai, Parvin Sultana, Hisanori Takenobu, Masayuki Haruta, Ryuichi P Sugino, Kyosuke Mukae, Shunpei Satoh, Tomoko Wada, Miki Ohira, Kiyohiro Ando, Takehiko Kamijo

Supplementary materials and methods

Cell lines

Human NB cell lines (NGP, NB-69 and SK-N-AS) were obtained from official cell banks (RIKEN Cell Bank, Tsukuba, Japan and ATCC, Manassas, VA, USA). The NGP and SK-N-AS cell lines were cultured in RPMI1640 (Wako, Osaka, Japan) supplemented with 10% heat-inactivated fetal bovine serum (FBS, Invitrogen, Carlsbad, CA, USA) and 100 μg/mL penicillin/streptomycin (Sigma-Aldrich, St. Louis, MO, USA). SK-N-FI cells were kindly provided by M. Schwab (Heidelberg, Germany) and grown in Dulbecco's modified Eagle's minimal essential medium (DMEM, Wako, Osaka, Japan) supplemented with 10% FBS, 100 μg/mL penicillin/streptomycin, and 1% MEM Non-Essential Amino Acids (Fujifilm Wako Pure Chemical Industries, Ltd., Osaka, Japan). Cultures were maintained at 37ºC under 5% CO_2 in air. The absence of mycoplasma contamination was confirmed using Mycoplasma PCR Detection set (TaKaRa).

ATRX CRISPR genome editing

CRISPR/Cas9 technology was used to generate *ATRX* KO cells. We designed three guide RNAs (gRNAs) against exons 4 and 5 of ATRX (Supplementary Fig. 1A, B). Regarding the Cas9 expression vector, we used the pHL-EF1a-SphcCas9-iP-A (Addgene ID: 60599)

vector to express human-codon optimized SpCas9 under the human EF1a promoter and puromycin resistance gene. Custom gRNA expression vectors were constructed in the pHL-H1-ccdB-mEF1a-RiH (Addgene ID: 60601) plasmid as previously described[1]. NGP and NB-69 cells were then electroporated with Cas9 and gRNA expression vectors using the NEPA21 electroporation system (Nepa Gene) under optimum conditions. Puromycin (0.5 μg/mL, 3 days)-selected cells were trypsinized and diluted in medium for single colony formation. Single colonies were selected and the *ATRX* KO status was examined using Sanger sequencing, Western blotting, and IF techniques.

In *ATRX* KO SK-N-AS cells, the same gRNAs targeting the exon of the *ATRX* gene (Supplementary Fig. 1A, B) were cloned into the LentiCRISPRv2 plasmid (Addgene Plasmid #52961). Standard laboratory protocols was used to produce lentiviral particles in HEK-293T cells. To generate stable cell lines, SK-N-AS cells were infected with the virus for each gRNA and empty LentiCRISPRv2 as a control. Single-cell colonies were selected, and their KO status was validated by Sanger sequencing, Western blotting, and IF.

Examination of off-target effects

Candidate off-target sites were searched using optimized CRISPR DESIGN (http://crispr.mit.edu). Among the candidates, a potential off-target locus was amplified

by PCR using the genomic DNA of established *ATRX* KO NGP and SK-N-AS cells as templates and nucleotide sequences were analyzed by Sanger sequencing. The results obtained are summarized in Supplementary Table S1. As shown in the table, no indels were observed at off-target loci in *ATRX* KO NGP or SK-N-AS cells.

Dominant-negative mutant p53 overexpression

The lentiviral vectors to express the mutant of p53 were pLenti6/V5-p53_R273H (Addgene ID: 22934) and pLenti6/V5-p53_R175H (Addgene ID: 22936), which were obtained from Bernard Futscher's laboratory (College of Pharmacy, University of Arizona, Tucson, AZ). The method to prepare the lentivirus was previously described[2]. Thereafter, 1×10^5 cells were seeded on each well of a six-well plate and infected using lentiviral-conditioned media with 4 μg/mL polybrene (Sigma-Aldrich). Stably infected cells were selected by a treatment with 4 μg/mL blasticidin (Wako Pure Chemical Industries, Ltd., Osaka, Japan). Transduced cells were analyzed by Western blotting.

Knockdown of FANCD2

pLKO.1-CMV-neomycin-based lentiviral vectors containing five sequence-verified shRNAs targeting human FANCD2 (RefSeqNM_033084.6) were obtained from the MISSION TRC (Human) shRNA library (Sigma-Aldrich) (Supplementary Table S2). We transduced these shRNAs using a lentiviral system. Two out of the five shRNAs

(TRCN0000082840: Sh-3, TRCN0000082841: Sh-4) were selected based on FANCD2 knockdown efficiency (Fig. 6A, B).

Cell proliferation and colony formation assays

Cells were seeded on 96-well plates in culture medium containing 10% FBS with three replicate wells for each group. At various time points, cell viability was measured by the water-soluble tetrazolium salt (WST-8) assay using Counting kit-8 (Dojindo, Kumamoto, Japan) according to the manufacturer's instructions. In the colony formation assay, single-cell suspensions were seeded on 6-well plates in triplicate. After 2-3 weeks, viable colonies were fixed with methanol (Methanol EMSURER ACS, Merck KgAa, Germany), stained with Giemsa (Giemsa's Azur Eosin Methylene blue, Merck KgAa), and the number of colonies in each well was calculated.

qPCR analysis

Total RNA was extracted from cultured cells using Trizol reagents (Life Technologies) or the RNeasy Mini kit (Qiagen). Reverse transcription was carried out by random primers and Superscript II (Invitrogen) according to the manufacturer's instructions. After reverse transcription, resultant cDNAs were subjected to a qPCR analysis as previously described[3] to measure the expression levels of *RTEL1*, *BLM*, *WRN*, *RECQL4*, *FANCJ*, and *FANCD2* mRNA. Expression levels were normalized to that of *GAPDH* and assessed

using the QuantStudio 7 Flex Real-Time PCR System (Applied Biosystems). Specific primer sequences for qPCR are listed in Supplementary Table S3.

Western blot analysis

Cells were washed three times with ice-cold PBS, and whole-cell extracts were prepared after lysis in EBC buffer[2]. EBC2 buffer containing 50 mM Tris·HCl, pH 8.0, 120 mM NaCl, 1 mM EGTA, and 0.5% Nonidet P-40 supplemented with 1 mM DTT, 10 mM β-glycerolphosphate, 10 mM NaF, 10 mM Na_3VO_4, 2 mM PMSF, and protease inhibitor mixture (Nacalai Tesque, Kyoto, Japan) was used for ATRX, ATM, and p-ATM immunoblotting. To reduce extract viscosity, 500 units/mL of benzonase (Sigma-Aldrich) and 50 mM $MgCl_2$ were added. Protein concentrations were measured using the Pierce BCA Protein Assay Kit (Thermo Fisher Scientific). Equal amounts of cell lysates were separated by SDS-PAGE and electrophoretically transferred onto Immobilon-P membranes (Millipore). Transferred membranes were blocked with 5% nonfat dry milk (Cell Signaling Technology) in 1× TBS and incubated with the appropriate primary antibodies at 4°C overnight followed by an incubation with horseradish peroxidase-conjugated anti-mouse or anti-rabbit secondary antibodies (Molecular & Biological Laboratories Co., Ltd.) at room temperature for 1 hour. Immunoreactive bands were visualized using the ECL clarity chemiluminescence kit (Bio-Rad Laboratories) with the

LAS-4000 luminescent image analyzer (Fuji Film, Tokyo, Japan). The primary and secondary antibodies used in the present study are shown in Supplementary Table S4.

IF

IF staining was performed as previously reported[4] with minor modifications. Cells grown on glass coverslips were fixed in 4% paraformaldehyde at 4°C for 20 min and permeabilized with 0.1% Triton X-100 at room temperature for 20 min. After an incubation with blocking solution containing 1% BSA plus 5% normal goat serum at room temperature for 1 h, cells were incubated with appropriate primary antibodies at 4°C overnight followed by an incubation with specific secondary antibodies listed in Supplementary Table S4. Nuclei were stained using ProLong Diamond Antifade Mountant with DAPI (Invitrogen), and fluorescent images were captured with a Keyence BZ-X710 fluorescence microscope. Regarding the phosphorylated H2AX (γH2AX) or G4 (1H6) staining of the positive control, cells were treated with doxorubicin (0.5 μg/mL) or CX-5461 (50 nM), respectively, for 24 hours following the protocol described above.

Immuno-FISH assay

After secondary antibody incubation in the IF technique (described above), cells were washed in blocking solution and 1× PBS, fixed with 4% paraformaldehyde for 10 min, and incubated with 10 mM glycine in H_2O at room temperature for 30 min. Coverslips

were washed three times in 1× PBS, air dried, denatured by setting the slides on a heating block at 80-85°C for 5 min in ready-to-use telG PNA probe/Cy3 (commercially available PNA FISH Kit/Cy3 K5326 from DAKO Glostrup Denmark), and hybridization was continued at 37°C overnight in dark moisturized chambers. Coverslips were then washed twice for 15 min with wash solution I (70% Formamide, 10 mM Tris·HCl, pH 7.4, and 0.1% BSA), followed by three 5-min washes with wash solution II (0.1 M Tris·HCl, pH 7.4, 0.15 M NaCl, and 0.08% Tween-20). Nuclear staining and image capturing were conducted as described above. Cells with γH2AX foci co-localizing with telomere DNA foci were scored as telomere dysfunction-induced foci (TIF) and counted from at least 100 nuclei.

Microarray analysis

A microarray analysis was performed as previously described[5]. Total RNA was extracted from Ctrl and two *ATRX* KO NGP clones (Fig. 2), and a microarray analysis was performed using the Agilent-072363 SurePrint G3 Human GEv3 8x60K Microarray G4851C (Agilent Technologies, Santa Clara, CA, USA). Differentially expressed genes (DEG) were set as fold changes of more than two ($p < 0.05$). Subsequent gene annotation was conducted by GeneSpringGX. GSEA was performed on DEG using GSEA software from the Broad Institute. Array data has been deposited in the Gene Expression Omnibus

(GEO) database (GEO accession: GSE183648).

C-circle assay and telomere qPCR

The amplification and quantitation of telomeric extrachromosomal circles (C-circles) in experimental cells were performed as previously described[6,7,8]. The assay was performed on each sample with and without phai29 polymerase. C-circle assay products were detected by telomere qPCR[9]. qPCR was performed in triplicate to compare telomere sequence contents to the single-copy gene VAV2, which has been used for NB cell lines[10]. The primer sequences used were as follows: telomere primers[9,11,12] forward 5′-CGGTTTGTTTGGGTTTGGGTTTGGGTTTGGGTTTGGGTT-3′ and reverse 5′-GGCTTGCCTTACCCTTACCCTTACCC TTACCCTTACCCT-3′; and VAV2 primers[10] forward 5′-TGGGCATGACTGAAGATGAC-3′ and reverse 5′-ATCTGCCCTCACCTTCTCAA-3′. PCR conditions were as follows: telomere: 95ºC for 15 min, 30 cycles of 95ºC for 15 s and 54ºC for 2 m, and VAV2: 95ºC for 5 min, 40 cycles of 95ºC for 15 s, 56ºC for 15 s, and 72ºC for 1 m. The relative C-circle level of a sample was expressed relative to those of SKN-FI cell lines with an arbitrary value of 196. The relative telomere content (T/S ratio) was assessed by qPCR of genomic DNA and quantified by comparing the amount of the telomere amplification product (T) to that of a single-copy gene (S).

Telomere FISH, PML immunofluorescence, and APB scoring

Telomere FISH and PML immunofluorescence are a combined method of indirect immunofluorescence with a FISH (Immuno-FISH) analysis, described in the Immuno-FISH assay. Regarding PML immunofluorescence, staining was conducted with the PML antibody (1:100, overnight) and Alexa fluor 488 goat anti-mouse antibody (1:1000, 1 h). In addition, telG PNA probe/Cy3 (commercially available PNA FISH Kit/Cy3 K5326 from DAKO Glostrup Denmark) was used for telomere FISH. Nuclei were stained using ProLong Diamond Antifade Mountant with DAPI (Invitrogen), and fluorescent images were captured with a Keyence BZ-X710 fluorescence microscope. Cells with PML foci co-localizing with telomere DNA foci were scored as positive for APBs and counted from at least 100 nuclei.

Statistical analysis

Data are shown as the mean ± standard deviation (SD). Statistical comparisons were performed by the Student's *t*-test or a one-way analysis of variance (ANOVA) followed by Dunnett's and Tukey's test, with $p < 0.05$ indicating significance. Regarding the WST assay, a two-way ANOVA followed by a multiple comparison Bonferroni post hoc test was used to compare differences between groups (*p < 0.05 and **p < 0.01).

Supplementary Fig. 1

A.

List of guide RNA target sequences	
**gRNA number**	**gRNA target sequence**
ATRX gRNA #1	TCGTGACGATCCTGAAGACT
ATRX gRNA #2	ACATACTTTGTTACAATTGA
ATRX gRNA #3	ACTATGCAGAGCTTGCCAAA

gRNA, guide RNA

B.

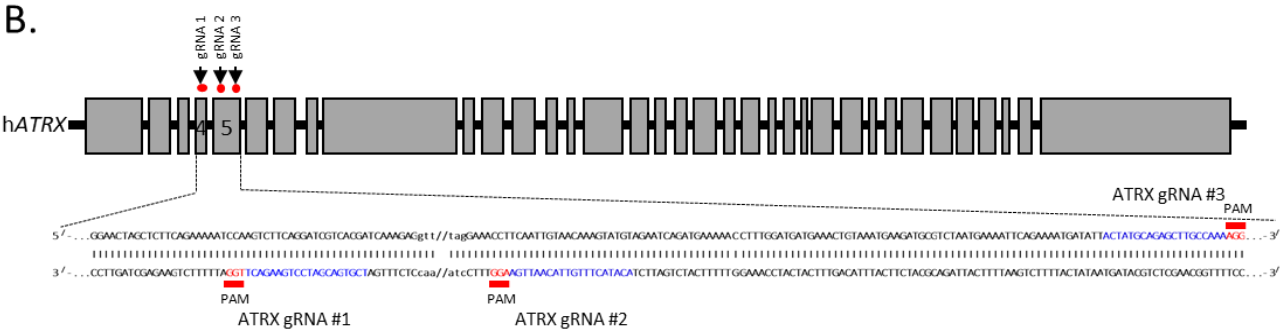

C.

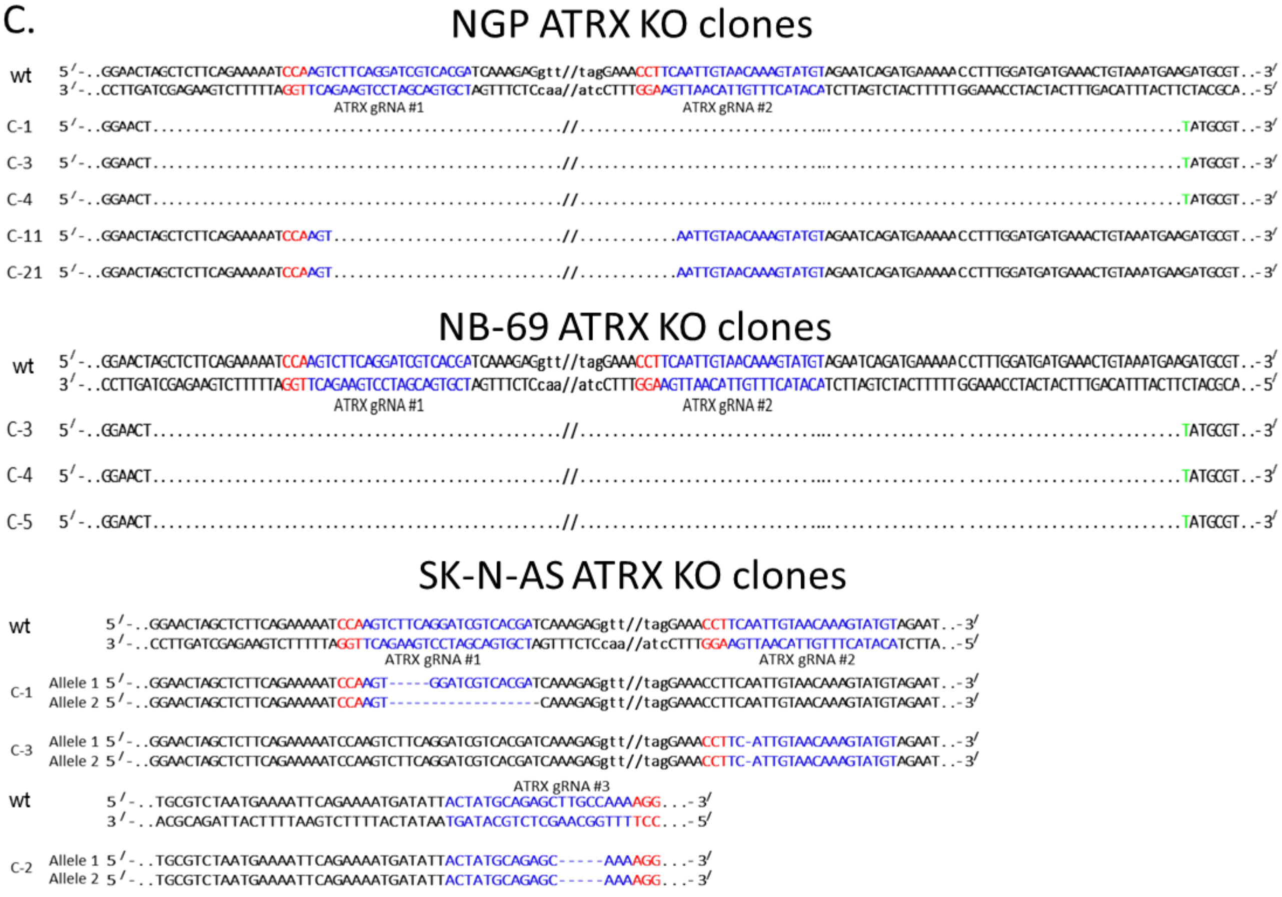

Supplementary Fig. 1: Targeted genomic deletion within the human *ATRX* locus.

A. List of guide RNA (gRNA) sequences used in the present study.

B. gRNA target sites within the human *ATRX* locus. Red dots indicate the location of three gRNA target sites. Enlarged view of exons 4 and 5 showing gRNA target sequences with the PAM sequence.

C. The sequencing results of *ATRX* KO clones in NGP (upper), NB-69 (middle) and SK-N-AS (lower) cells. All clones confirmed the frameshift deletion. wt, wild type.

Supplementary Fig. 2

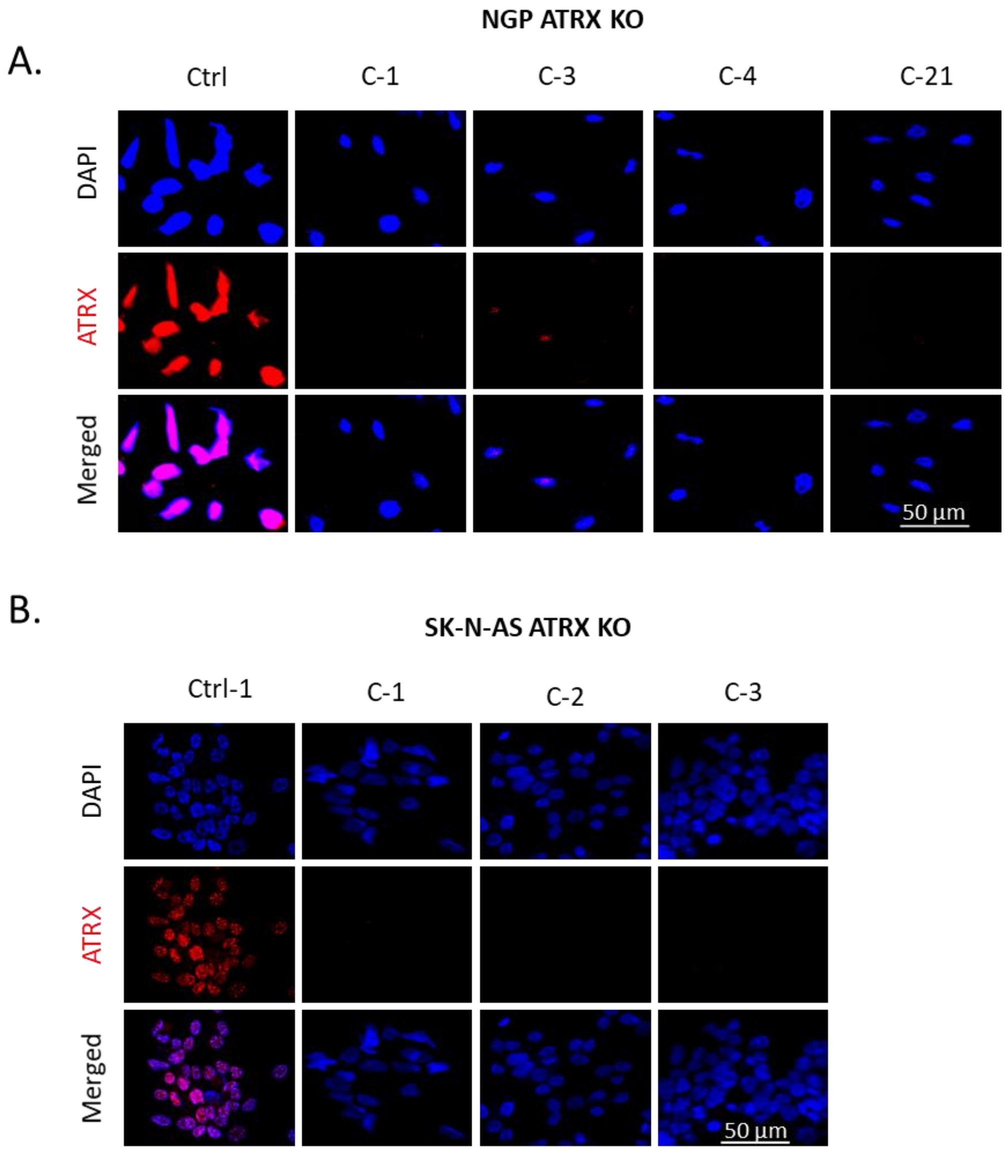

Supplementary Fig. 2: Confirmation of *ATRX* KO in NGP and SK-N-AS cells.

A, B. IF detection of ATRX in Ctrl and *ATRX* KO NGP (A) and SK-N-AS (B) cells showing the loss of ATRX expression.

Supplementary Fig. 3

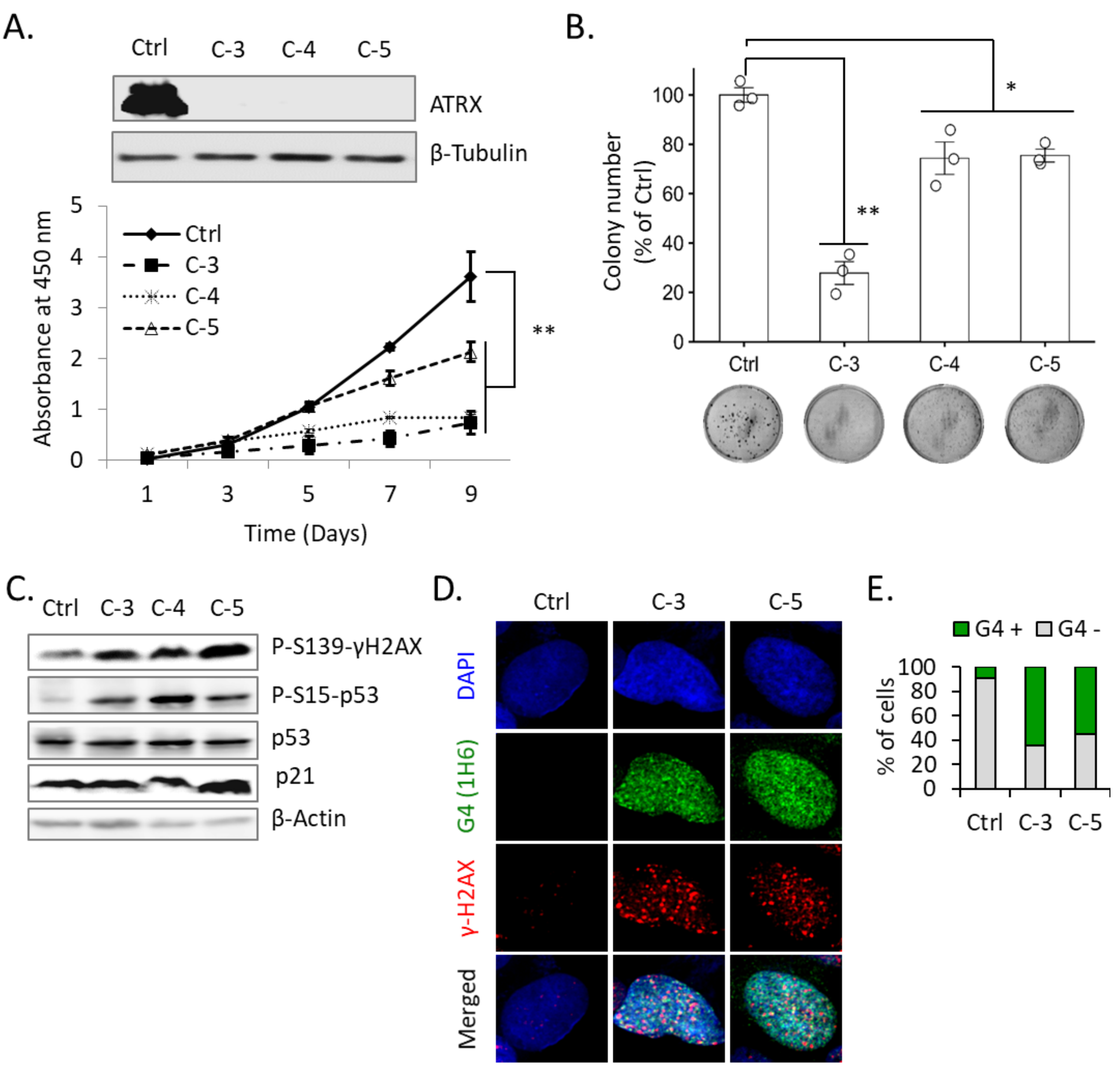

Supplementary Fig. 3: ATRX loss-induced DDRs in *MYCN* single-copy and *TP53* wt NB-69 cells.

A. Western blots show the depletion of ATRX protein expression in cell lysates prepared

from Cas9 control (Ctrl) and *ATRX* KO (C-3, C-4, and C-5) NB-69 cells. β-Tubulin was used as a loading control. Lower panel, growth curves show that viability was lower in *ATRX* KO NB-69 cells than in Ctrl cells. Data are expressed as means ± standard deviation (SD), N = 3. A two-way ANOVA followed by a multiple comparison Bonferroni post hoc test was used to compare differences between groups (**$p < 0.01$).

B. Clonogenic assay of Ctrl and *ATRX* KO NB-69 cells demonstrating the weaker proliferative abilities of KO cells than Ctrl cells. Lower panel, representative images for clonogenic formation are shown. Error bars represent SD from three technical replicates. *$p < 0.05$ and **$p < 0.01$; A one-way ANOVA with Dunnett's and Tukey's test were used for statistical analyses.

C. Representative immunoblots for the expression of the indicated proteins in cell lysates prepared from Ctrl and *ATRX* KO NB-69 cells**.** *ATRX* KO NB-69 cells showed a modest activation in the p53-pathway. β-Tubulin was used as a loading control.

D, E. Coimmunofluorescence staining of ATRX intact (Ctrl) and *ATRX* KO NB-69 cells with the anti-G-quadruplex (G4) antibody, 1H6 and anti-γH2AX. (E) Quantification of G4 + cells among 100 cells analyzed in (D).

Supplementary Fig. 4

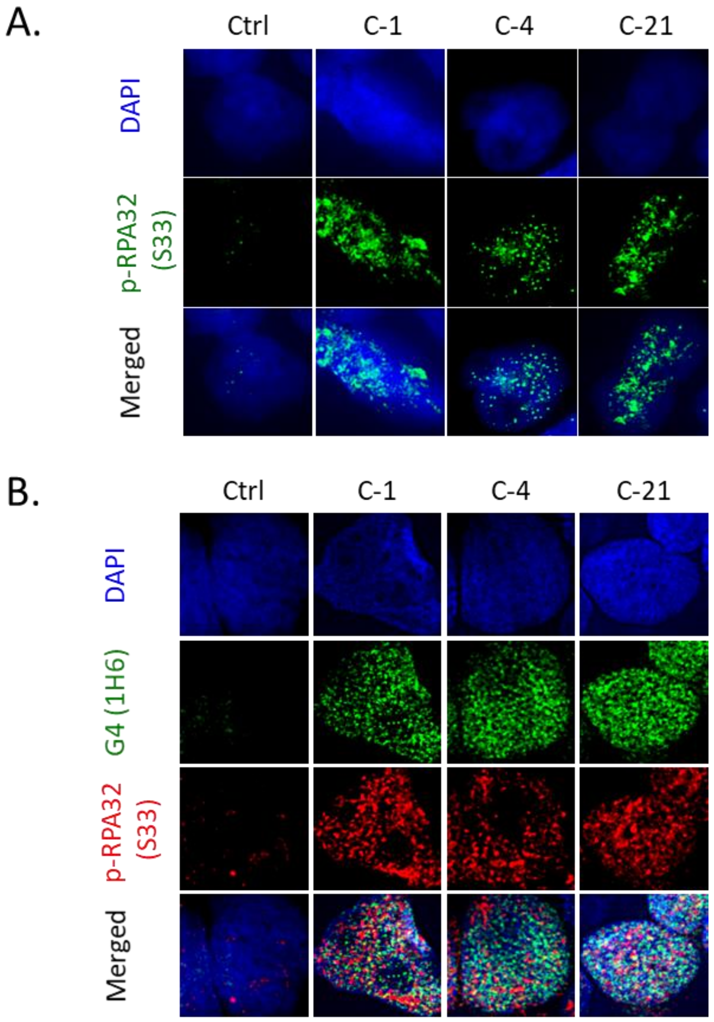

Supplementary Fig. 4: ATRX loss-induced replication stress in *TP53* wt *ATRX* KO NGP cells.

A. p-RPA32 (S33) IF revealed strongly increased replication stress in NGP cells after the deletion of ATRX. Nuclear staining is blue and p-RPA32 (S33) staining is green.

B. Coimmunofluorescence for G4 (1H6) and p-RPA32 (S33) reveals the colocalization in *ATRX* KO cells.

Supplementary Fig. 5

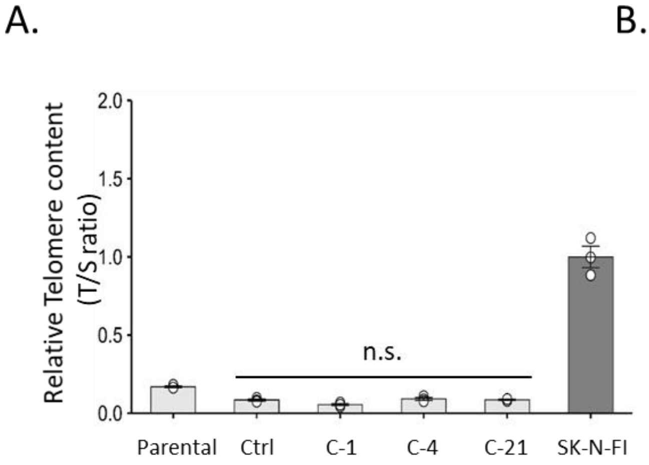

B. C-circle analysis

	Telomere content	SD	C-circle	SD
Parental	1.4	0.048	0.0	0.054
Ctrl	1.0	0.018	0.2	0.009
C-1	1.2	0.059	-0.2	0.036
C-4	2.1	0.047	-0.1	0.072
C-21	1.1	0.030	0.2	0.011
SK-N-FI	14.0	0.633	196	6.257

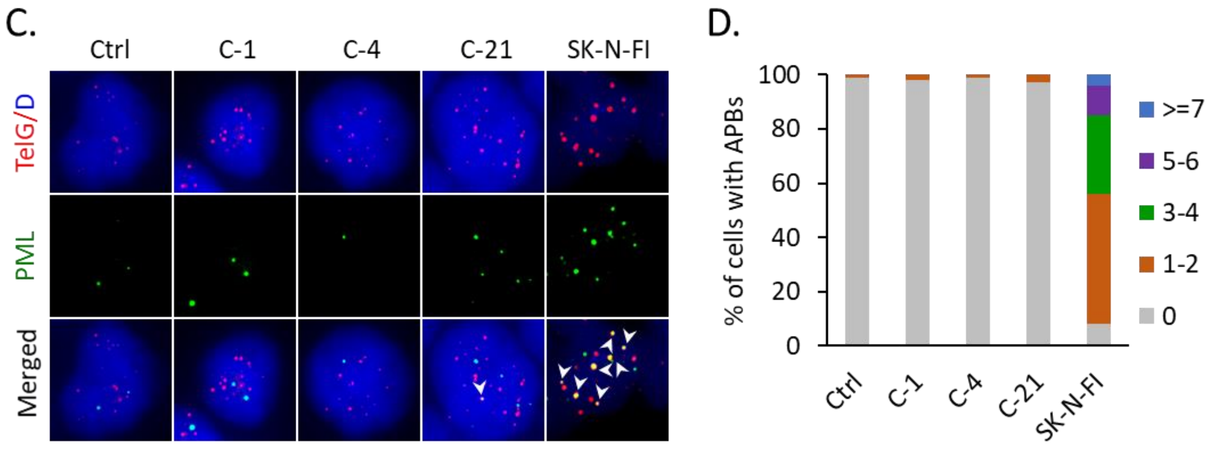

Supplementary Fig. 5: ATRX deficiency in itself is not sufficient to induce the alternative lengthening of telomeres (ALT) phenotype in NGP cells.

A. Relative telomeric DNA contents (T/S ratio) assessed by qPCR in parental, Ctrl, or *ATRX* KO NGP and ALT^{+} SK-N-FI cells. Using this qPCR method, the relative telomeric content is the ratio of a PCR reaction product from the same sample using specific primers

for telomeres and single copy genes (T/S ratio). Error bars denote SD; n = 3 (experimental triplicates); one-way ANOVA with Dunnett's and Tukey's test; n.s. not significant.

B. C-circle assay of parental, Ctrl, or *ATRX* KO NGP and ALT^{+} SK-N-FI cells. The relative telomere DNA content of a sample was the normal telomere content obtained from the C-circle assay without phai29 polymerase and was relative to the telomere content of the ALT^{+} SK-N-FI cell line with arbitrary (AU) values of 14.0. C-circle levels were calculated relative to that in the ALT^{+} SK-N-FI cell line (designated to be 196 AU). As previously described, a sample was considered to be ALT^{+} when the relative C-circle level was ≥7.5 and the relative telomere content >12[6,8].

C. Representative images showing the localization of PML and telomeres in Ctrl and *ATRX* KO NGP cells. PML were detected by IF (green) and telomeres by FISH using the TelG PNA probe. ALT^{+} SK-N-FI cells used as a positive control. Arrows indicate an example of ALT-associated PML bodies (APBs).

D. Cells were scored for APBs in Ctrl and *ATRX* KO clones. Approximately 100 cells were divided into 5 groups (0, 1 or 2, 3 or 4, 5 or 6, and ≥7) based on the numbers of APB^{+} telomeres in each isogenic cell.

Supplementary Fig. 6

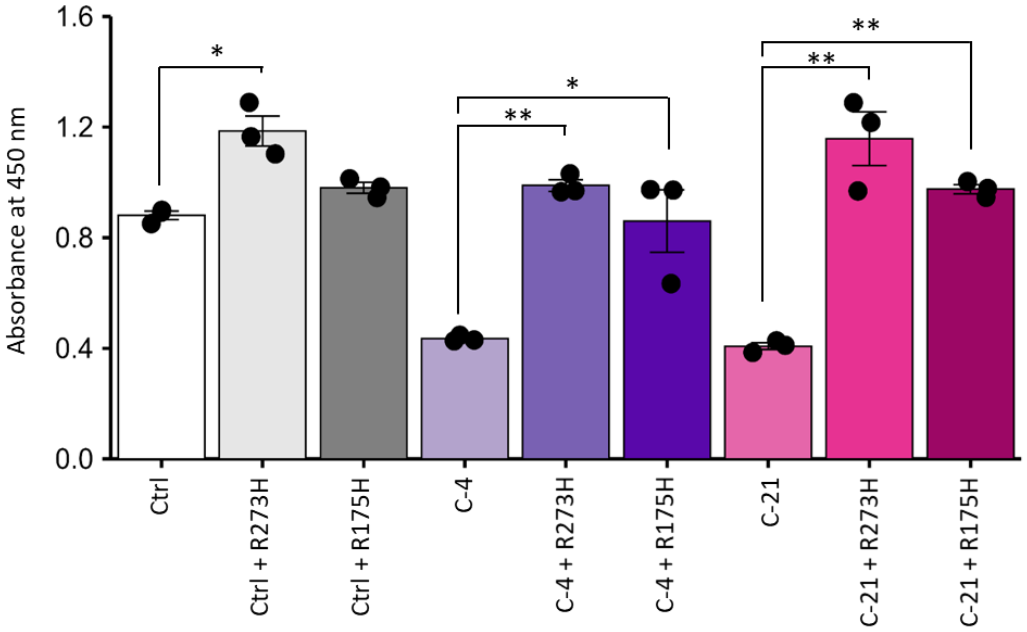

Supplementary Fig. 6: Mutant p53 (R273H and R175H) inhibits the ability of wild-type p53 to suppress cell proliferation after the deletion of ATRX. The R273H or R175H dominant-negative variants of p53 were stably overexpressed in Ctrl or *ATRX* KO NGP cells (Fig. 3A). The proliferation rates of *TP53* wt Ctrl or *ATRX* KO with p53-inactivated Ctrl or *ATRX* KO NGP cells. The bars represent means with SDs from three experimental replicates. Statistical significance was calculated using two-tailed *t*-test. *$p < 0.05$ and **$p < 0.01$.

Supplementary Fig. 7

A.

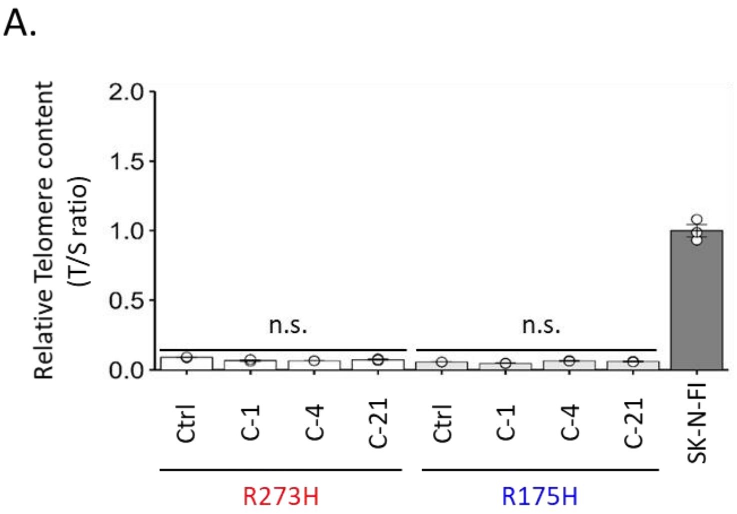

B.

		Telomere content	SD	C-circle	SD
R273H	Ctrl	1.5	0.096	0.9	0.030
	C-1	1.3	0.044	0.1	0.154
	C-4	2.0	0.150	-0.3	0.056
	C-21	1.8	0.066	-0.4	0.036
R175H	Ctrl	2.1	0.107	-1.2	0.069
	C-1	0.8	0.030	0.3	0.045
	C-4	1.3	0.013	0.2	0.062
	C-21	1.1	0.025	0.5	0.033
	SK-N-FI	14.0	0.823	196	3.780

C.

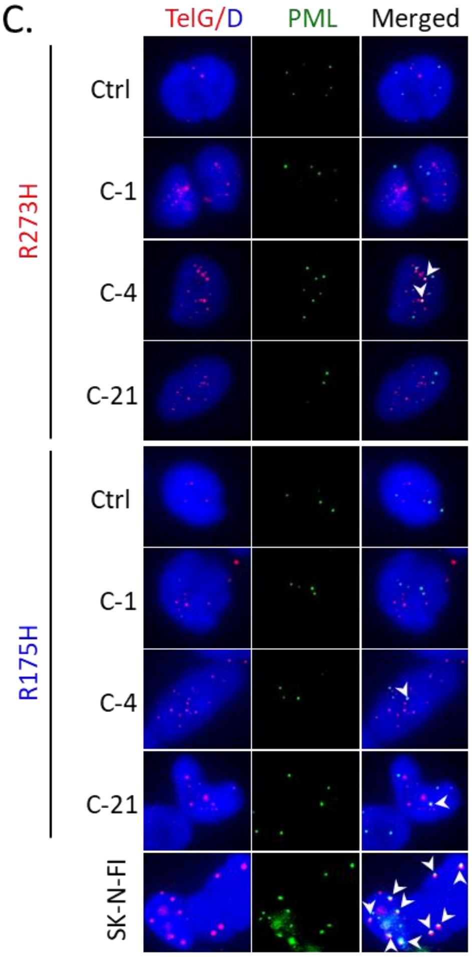

D.

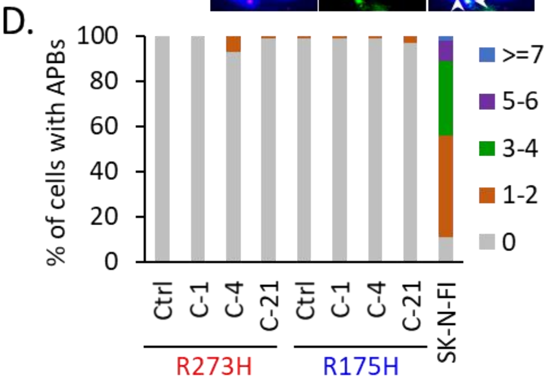

Supplementary Fig. 7: Concomitant p53 inactivation and ATRX loss are not sufficient

for the induction of ALT characteristics.

A. Relative telomeric DNA contents in p53-inactivated Ctrl or *ATRX* KO NGP and ALT^{+} SK-N-FI cells were assessed as described in Supplementary Fig. 4A. Error bars denote SD; n = 3 (experimental triplicates); n.s. not significant.

B. C-circle levels in *p53*-inactivated Ctrl or *ATRX* KO NGP and ALT^{+} SK-N-FI cells. Quantification was performed as described in Supplementary Fig. 4B.

C. Representative images of telomere FISH (red) and PML IF (green) from *p53*-inactivated Ctrl or *ATRX* KO NGP cells. Arrows show an example of ALT-associated PML bodies (APBs). ALT^{+} SK-N-FI cells were used as a positive control.

D. Cells were scored for APBs and ~100 cells were divided into 5 groups based on the numbers of APB^{+} telomeres in each cell.

Supplementary Fig. 8

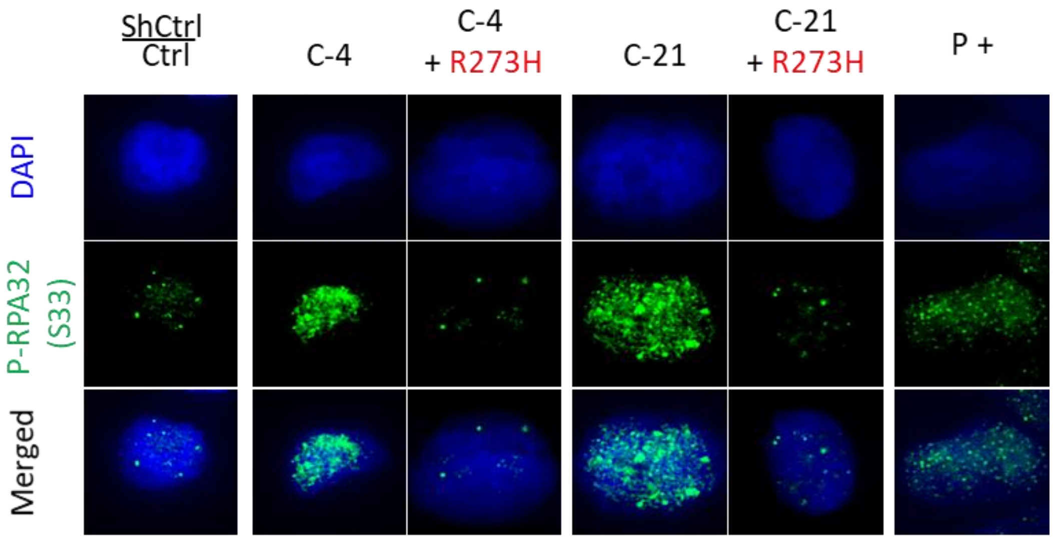

Supplementary Fig. 8: IF staining of Ser33 phospho RPA32 in indicated cells. Dominant-negative p53 mutants mediated p53 inactivation reduced the replication stress induced by the deletion of ATRX in NGP cells. As a positive control (P+), parental NGP cells were treated with doxorubicin (0.5 µg/mL) for 24 hours and stained with the anti-p-RPA32 (S33) antibody.

Supplementary Fig. 9

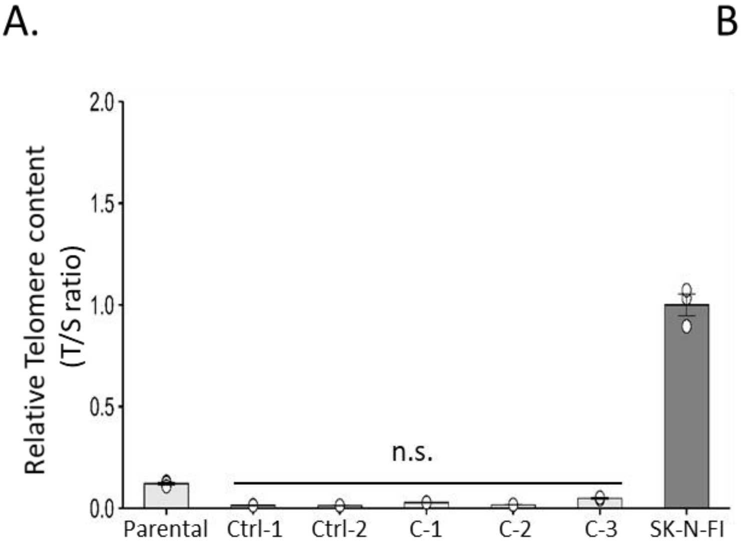

B.

	Telomere content	SD	C-circle	SD
Parental	3.4	0.077	0.9	0.182
Ctrl-1	0.8	0.029	0.0	0.060
Ctrl-2	0.7	0.018	-0.1	0.020
C-1	1.2	0.045	-0.2	0.072
C-2	0.8	0.017	0.5	0.077
C-3	2.2	0.066	-0.9	0.025
SK-N-FI	14.0	0.797	196	1.532

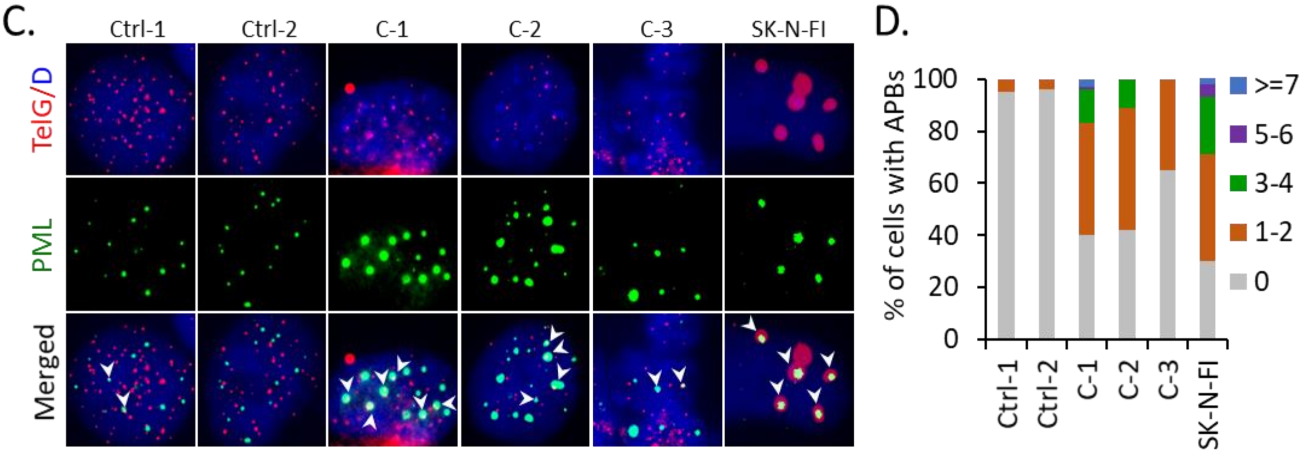

Supplementary Fig. 9: ALT phenotypes in *ATRX* KO SK-N-AS cells.

A. Relative telomeric DNA contents, measured by telomere qPCR for parental, Ctrl, or *ATRX* KO SK-N-AS cells, were assessed as described in Supplementary Fig. 4A. ALT^+ SK-N-FI cells were used as a positive control. Error bars denote SD; n = 3 (experimental triplicates); n.s. not significant.

B. C-cycle assay of parental, Ctrl, or *ATRX* KO SK-N-AS cells. C-circle levels were calculated relative to those in the ALT^+ SK-N-FI cell line (designated to be 196 AU).

C. APBs (co-localization of telomeric foci (red) and PML nuclear bodies (green), indicated by arrows) were frequently found in ALT^+ SK-N-FI cells. *ATRX* KO SK-N-AS cells also showed positivity for APBs to a certain extent.

D. APB positivity was scored and ~100 cells were divided into 5 groups based on the numbers of APB+ telomeres in each cell.

Supplementary Fig. 10

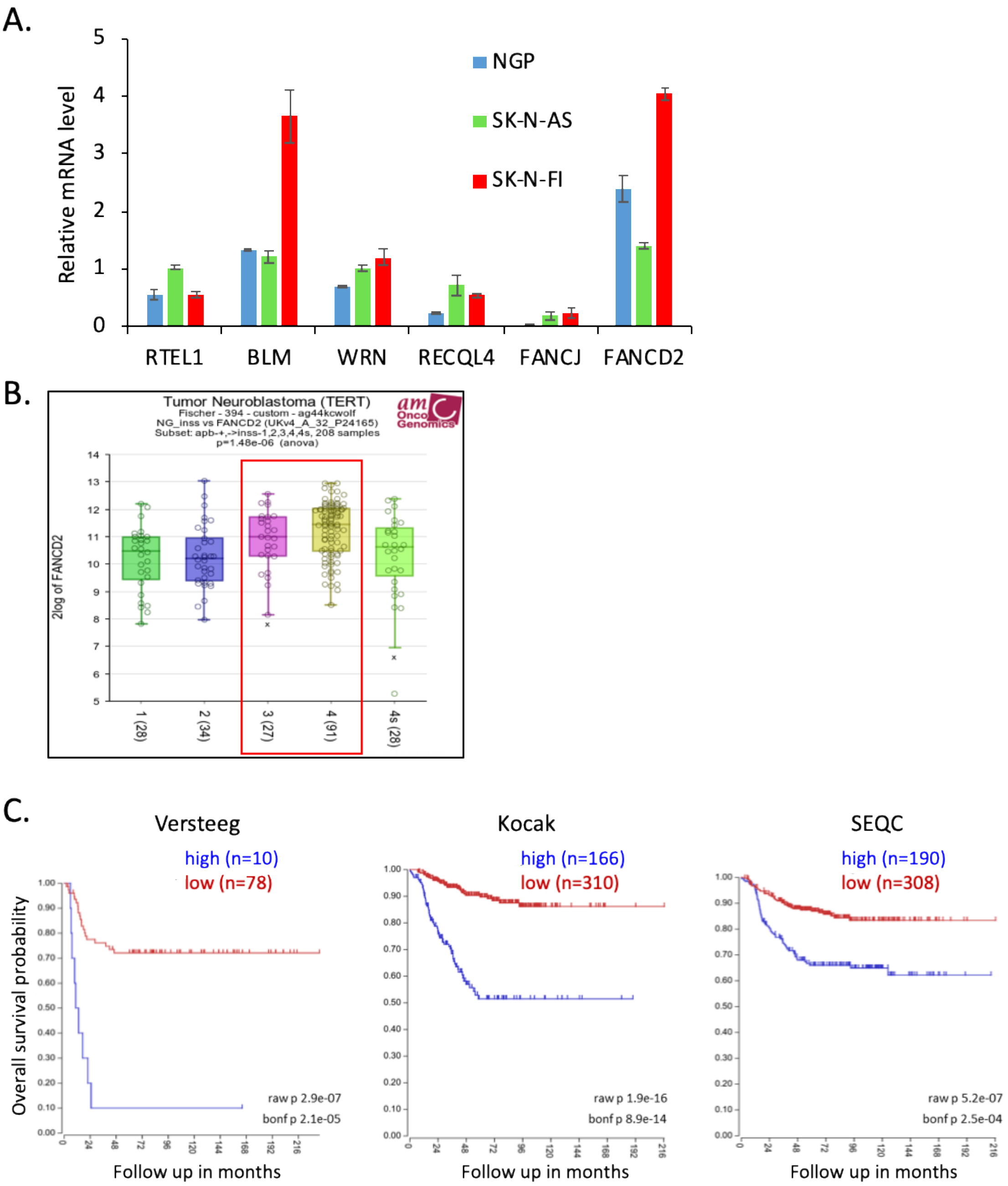

Supplementary Fig. 10: Relationship between *FANCD2* expression and NB patient survival

A. RT-PCR analysis of G4 -resolving helicase genes and *FANCD2* in NGP, SK-N-AS, and SK-N-FI cells. Relative expression normalized to GAPDH, Mean ± SD (n=3).

B. *FANCD2* expression patterns in different INSS groups.

C. Kaplan-Meier curve based on *FANCD2* expression.

Supplementary Fig. 11

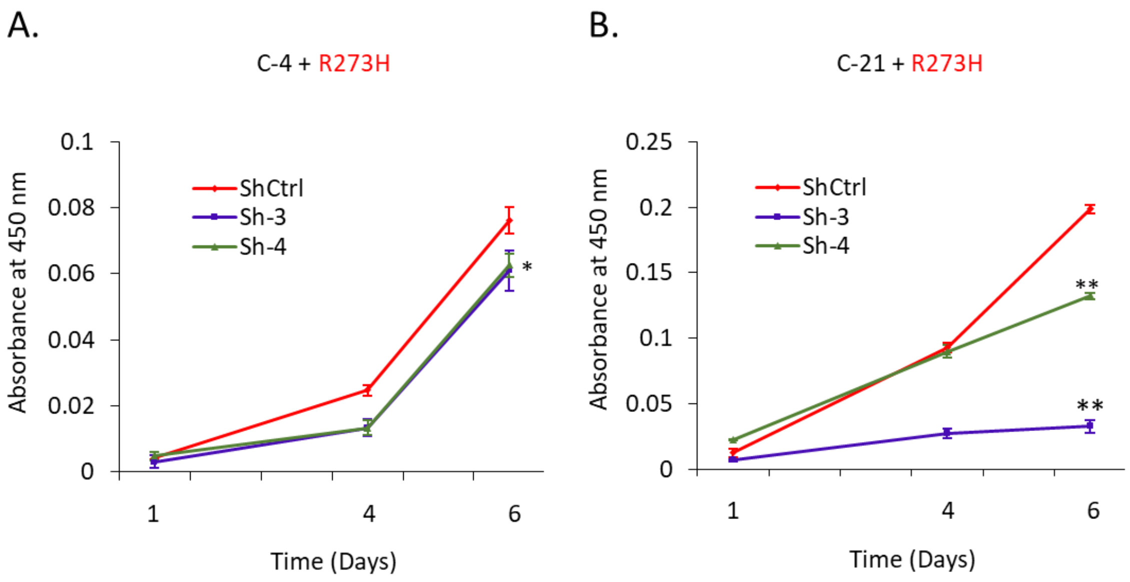

Supplementary Fig. 11: Up-regulated FANCD2 promotes the survival of ATRX-deficient cells with p53 inactivation.

A, B. Proliferation rates of p53-inactivated *ATRX* KO NGP cells (C-4 +R273H and C-21 +R273H) infected with ShCtrl and FANCD2 shRNAs. Data are expressed as means ± standard deviation (SD), N = 3. A two-way ANOVA followed by a multiple comparison Bonferroni post hoc test was used to compare differences between groups (*$p < 0.05$ and **$p < 0.01$).

Supplementary Fig. 12

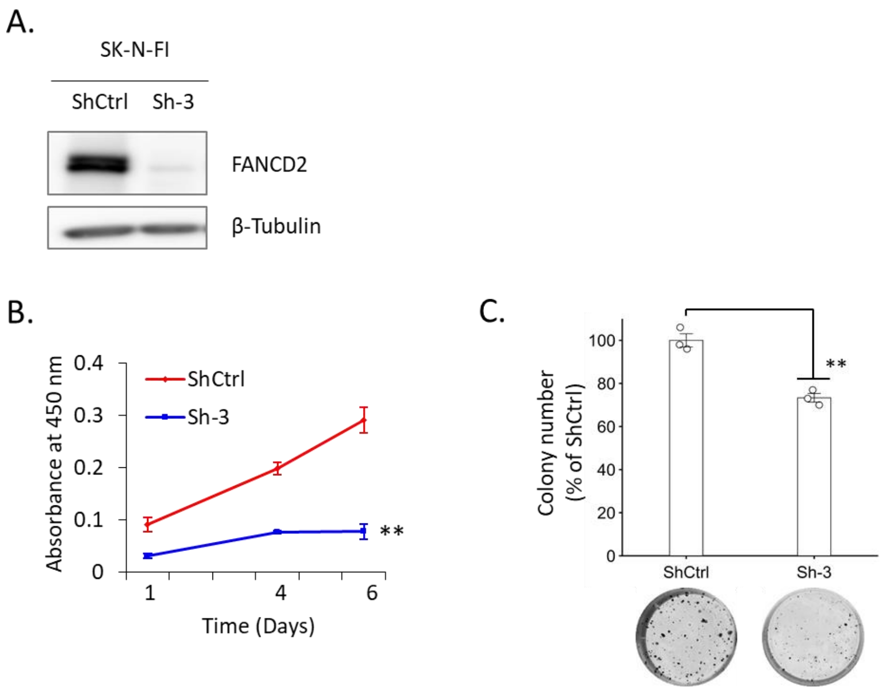

Supplementary Fig. 12: FANCD2 knockdown with shRNA interference reduces the cell viability in SK-N-FI cells.

A. FANCD2 protein depletion by shRNA was confirmed by immunoblotting.

B. Growth curves show that viability was lower in FANCD2 knockdown SK-N-FI cells than in ShCtrl cells. Data are expressed as means ± standard deviation (SD), N = 3. Two-tailed *t*-test, **$p < 0.01$.

C. Clonogenic assay demonstrating the weaker proliferative abilities of FANCD2 knockdown SK-N-FI (Sh-3) cells than in ShCtrl cells. Lower panel, representative images for clonogenic formation are shown. Error bars represent SD from three technical replicates. Two-tailed *t*-test, **$p < 0.01$.

Supplementary Table S1

Off-target analysis of gRNA1, gRNA2 and gRNA3 in NGP and SK-N-AS *ATRX* KO cells. Blue colored letters indicate the PAM sequence. Red highlighted letters indicate the differences of the gRNA with the target sequence. Indel column shows the detected off target results. No off-target edits were detected in any loci tested for gRNA #1 (n=5), gRNA #2 (n=4) and gRNA #3 (n=3).

Genomic location	Locus details	Sequence	Indel
gRNA #1			
On-target	Exon: ATRX	TCGTGACGATCCTGAAGACT TGG	
Off-target			
Chr12: 192493-192515	Exon: SLC6A12	TCATCACCCTCCTGAAGACT CGG	no
Chr1: 113124438-113124460	Exon: LRIG2	TCAGGACCATTCTGAAGACT AGG	no
Chr1: 111981829-111981851	Exon: KCND3	ACTTGAAGATCCTGAAGACG CGG	no
Chr3: 57559716-57559738	Exon: PDE12	TCCAGAGGATCATGAAGACT GGG	no
Chr7: 120275508-120275530	Exon: KCND2	TCCTGAAGACCCGGAAGACT CGG	no
gRNA #2			
On-target	Exon: ATRX	ACTATGCAGAGCTTGCCAAA AGG	
Off-target			
Chr11: 8436099-8436121	Exon: STK33	TCTCTTCTGAGCTTGCCAAA AAG	no
Chr1: 1387858-1387880	Exon: cyclin L2	CCTGTTCACAGCTTGCCAAA GGG	no
Chr7: 149103630-149103652	Exon: ZNF425	ACATTGCAGTGCATGCCAAA GAG	no
Chr3: 49325808-49325830	Exon: USP4	ACTTTGCAGTGCTTGACAAA CAG	no
gRNA #3			
On-target	Exon: ATRX	ACATACTTTGTTACAATTGA AGG	
Off-target			
Chr15: 75359267-75359289	Exon: MAN2C1	GGATACTTTCTTACAACTGA GGG	no
Chr4: 79902291-79902313	Exon: ANTXR2	CCATAGTATGTTACAATTGC TAG	no
Chr16: 1580685-1580707	Intron: IFT140	ACAGGCTTTGTCTCAATTGA GAG	no

Supplementary Table S2

Targeting sequences of shRNAs against human FANCD2 used in this study.

shRNA	Clone ID (Sigma)	Clone Name	Target sequence
Sh-1	TRCN0000082838	NM_033084	CCGGCGTCTATTAGATTGGAGGATTCTCGAG AATCCTCCAATCTAATAGACGTTTTTG
Sh-2	TRCN0000082839	NM_033084	CCGGGCGTCCATTACTTGCAGAATTCTCGAG AATTCTGCAAGTAATGGACGCTTTTTG
Sh-3	TRCN0000082840	NM_033084	CCGGCGACTCATTGTCAGTCAACTACTCGAG TAGTTGACTGACAATGAGTCGTTTTTG
Sh-4	TRCN0000082841	NM_033084	CCGGCCTCATACTGTTACTGCTATTCTCGAG AATAGCAGTAACAGTATGAGGTTTTTG
Sh-5	TRCN0000082842	NM_033084	CCGGCCACTGAGGTATCTCTACAAACTCGAG TTTGTAGAGATACCTCAGTGGTTTTTG

Supplementary Table S3

List of primer sequences used for qPCR analysis in this study.

Gene name	Forward primer sequences (5′→3′)	Reverse primer sequences (5′→3′)
hRTEL1	CCTATCCTGTCATGGAGAAGAGC	TCTCGGAGAAGCTGCCTTTGCT
hBLM	TGTTACACCACCCCAAAGTCA	GGAGGCAAATCAGTCTTTACTGT
hWRN	TGCAGCCATTTCTTGTCAAA	GAAGGACAGTAGATGATTGTTGGA
hRECQL4	TCACAGTGAGGTCCCAGATT	CTGACTTCTTGGAAGGCTGA
hFANCJ	CAATGCCCGTGCTGTCA	ATCTGCTGCCGTACCCATTTA
hFANCD2	AGACTGTCAAAATCTGAGGATAAAGAGA	TGGTTGCTTCCTGGTTTTGG
hGAPDH	ATGGAAATCCCATCACCATCTT	CGCCCCACTTGATTTTGG

Supplementary Table S4

List of antibodies used in this study.

Primary antibodies	Merchant, Catalog number	Species, type	Application
ATRX	Santa Cruz (H-300): sc-15408	Rabbit, Polyclonal	WB (1:1000), IF (1:100)
Phospho-γH2AX Ser 139	Millipore (JBW301), 05-636	Mouse, Monoclonal	WB (1:4000), IF (1:500)
Phospho-Histone H2AX Ser 139	Cell signaling, 2577	Rabbit	IF (1:100)
Phospho–KAP1 Ser 824	Bethyl Laboratories, A300-767A	Rabbit, Polyclonal	WB (1:2000)
KAP1	Bethyl Laboratories, A300-274A	Rabbit, Polyclonal	WB (1:1000)
Phospho–Chk1 Ser 345	Cell signaling, 13303	Rabbit, Polyclonal	WB (1:1000)
Chk1	Santa Cruz (G4): sc-8408	Mouse, Monoclonal	WB (1:1000)
Phospho–Chk2 Thr 68	Cell signaling (C13C1), 2197	Rabbit, Monoclonal	WB (1:1000)
Chk2	Merck (Clone 7), 05-649	Mouse, Monoclonal	WB (1:1000)
Phospho–RPA32 Ser 33	Bethyl Laboratories, A300-246A	Rabbit, Polyclonal	WB (1:5000), IF (1:1000)
RPA32	Bethyl Laboratories, A300-244A	Rabbit, Polyclonal	WB (1:2000)
Phospho–ATM Ser 1981	Millipore 10H11.E12, 05-740	Mouse, Monoclonal	WB (1:1000)
ATM	Santa Cruz (2C1): sc-23921	Mouse, Monoclonal	WB (1:1000)
Phospho–p53 Ser 15	Cell signaling, 9284	Rabbit, Polyclonal	WB (1:1000)
p53	Santa Cruz (DO-1): sc-126	Mouse, Monoclonal	WB (1:1000)
p21	Santa Cruz (187): sc-817	Mouse, Monoclonal	WB (1:1000)
V5	ThermoFisher Scientific, R960-25	Mouse, Monoclonal	WB (1:5000)
G4 (1H6)	EMD Millipore, MABE1126	Mouse, Monoclonal	IF (1:100)
FANCD2	Novus Biologics, NB100-182	Rabbit, Polyclonal	WB (1:3000)
PML	Santa Cruz (PG-M3): sc-966	Mouse, Monoclonal	IF (1:100)
β-Actin	Sigma-Aldrich (20-33), A5060	Rabbit, Polyclonal	WB (1:3000)
β-Tubulin	Wako, 10G10	Mouse, Monoclonal	WB (1:2000)
**Secondary antibodies**	**Merchant, Catalog number**	**Species, type**	**Application**
Anti-Rabbit IgG (H+L) HRP	MBL, 458	Rabbit, Polyclonal	WB (1:6000)
Anti-Mouse IgG (H+L) HRP	MBL, 330	Mouse, Polyclonal	WB (1:6000)
Alexa fluor 488 F(ab')2-goat anti-rabbit IgG (H+L)	Molecular probe, A11070	Rabbit, Polyclonal	IF (1:1000)
Alexa fluor 594 F(ab')2-goat anti-rabbit IgG (H+L)	Molecular probe, A11072	Rabbit, Polyclonal	IF (1:1000)
Alexa fluor 488 F(ab')2-goat anti-mouse IgG (H+L)	Molecular probe, A11017	Mouse, Polyclonal	IF (1:1000)

Supplementary References:

1 Li HL, Gee P, Ishida K, Hotta A. Efficient genomic correction methods in human iPS cells using CRISPR-Cas9 system. *Methods* 2016; **101**: 27–35.

2 Ochiai H, Takenobu H, Nakagawa A, Yamaguchi Y, Kimura M, Ohira M *et al.* Bmi1 is a MYCN target gene that regulates tumorigenesis through repression of KIF1BB and TSLC1 in neuroblastoma. *Oncogene* 2010; **29**: 2681–2690.

3 Akter J, Takatori A, Hossain MS, Ozaki T, Nakazawa A, Ohira M *et al.* Expression of NLRR3 orphan receptor gene is negatively regulated by MYCN and Miz-1, and its downregulation is associated with unfavorable outcome in neuroblastoma. *Clin Cancer Res* 2011; **17**: 6681–6692.

4 Akter J, Takatori A, Islam MS, Nakazawa A, Ozaki T, Nagase H *et al.* Intracellular fragment of NLRR3 (NLRR3-ICD) stimulates ATRA-dependent neuroblastoma differentiation. *Biochem Biophys Res Commun* 2014; **453**: 86–93.

5 Li Z, Takenobu H, Setyawati AN, Akita N, Haruta M, Satoh S *et al.* EZH2 regulates neuroblastoma cell differentiation via NTRK1 promoter epigenetic modifications. *Oncogene* 2018; **37**: 2714–2727.

6 Henson JD, Cao Y, Huschtscha LI, Chang AC, Au AYM, Pickett HA *et al.* DNA

C-circles are specific and quantifiable markers of alternative- lengthening-of-telomeres activity. *Nat Biotechnol* 2009; **27**: 1181–1185.

7 Farooqi AS, Dagg RA, Choi LMR, Shay JW, Reynolds CP, Lau LMS. Alternative lengthening of telomeres in neuroblastoma cell lines is associated with a lack of MYCN genomic amplification and with p53 pathway aberrations. *J Neurooncol* 2014; **119**: 17–26.

8 Dagg RA, Pickett HA, Neumann AA, Napier CE, Henson JD, Teber ET *et al.* Extensive Proliferation of Human Cancer Cells with Ever-Shorter Telomeres. *Cell Rep* 2017; **19**: 2544–2556.

9 Henson JD, Lau LM, Koch S, Martin La Rotta N, Dagg RA, Reddel RR. The C-Circle Assay for alternative-lengthening-of-telomeres activity. *Methods* 2017; **114**: 74–84.

10 Lau LMS, Dagg RA, Henson JD, Au AYM, Royds JA, Reddel RR. Detection of alternative lengthening of telomeres by telomere quantitative PCR. *Nucleic Acids Res* 2013; **41**: e34–e34.

11 Callicott RJ, Womack JE. Real-time PCR assay for measurement of mouse telomeres. *Comp Med* 2006; **56**: 17–22.

12 O'Callaghan NJ, Fenech M. A quantitative PCR method for measuring absolute

telomere length. *Biol Proced Online* 2011; **13**: 3.
